# Ultrathin transition metal oxychalcogenide catalysts for oxygen evolution in acidic media

**DOI:** 10.1038/s44160-024-00694-3

**Published:** 2025-01-02

**Authors:** Wenshuo Xu, Yao Wu, Shibo Xi, Yan Wang, Ye Wang, Yuxuan Ke, Lingtong Ding, Xiao Wang, Jieun Yang, Wenjing Zhang, Kian Ping Loh, Feng Ding, Zheng Liu, Manish Chhowalla

**Affiliations:** 1 Department of Materials Science and Metallurgy, University of Cambridge, Cambridge, UK; 2 School of Materials Science and Engineering, Nanyang Technological University, Singapore, Singapore; 3 Institute of Sustainability for Chemicals, Energy and Environment, Agency for Science, Technology and Research (A*STAR), Singapore, Singapore; 4 State Key Laboratory of Radio Frequency Heterogeneous Integration, Shenzhen University, Shenzhen, China; 5 Faculty of Materials Science and Energy Engineering, Shenzhen University of Advanced Technology, Shenzhen, China; 6 Institute of Technology for Carbon Neutrality, Shenzhen Institute of Advanced Technology, Chinese Academy of Sciences, Shenzhen, China; 7 Guangdong Provincial Key Laboratory of Functional Oxide Materials and Devices, Department of Materials Science and Engineering, Southern University of Science and Technology, Shenzhen, China; 8 Department of Chemistry, Kyung Hee University, Seoul, Republic of Korea; 9 Department of Chemistry, National University of Singapore, Singapore, Singapore

**Keywords:** Two-dimensional materials, Synthesis and processing

## Abstract

Two-dimensional transition metal dichalcogenides (TMDs) exfoliated from bulk layered materials possess interesting properties. Most transition metal oxides are not layered and therefore cannot be exfoliated. Here we report the synthesis of a family of ultrathin materials—transition metal oxychalcogenides (TMOCs)—and demonstrate their unique properties. Two-dimensional TMOCs (MX_*x*_O_*y*_, M = group IV or V transition metal, X = chalcogen, O = oxygen; *x*, *y* = 0–2) from bulk transition metal dichalcogenides (MX_2_) have been fabricated using tetrabutylammonium intercalation. The stoichiometry of TMOCs can be adjusted, which enables control of their optical bandgaps and tunability of electrical conductivity by more than eight orders of magnitude. By tuning the chalcogen-to-oxygen ratio along with local atomic structure in TMOCs, it is possible to impart unexpected properties. For example, in contrast to conventional TMDs, the hybrid structure of TMOCs renders them surprisingly stable and electrochemically active in strong acids, allowing them to be used as proof-of-concept catalysts for the oxygen evolution reaction at pH ≈ 0. The HfS_0.52_O_1.09_ catalyst shows high mass activity (103,000 A g^−1^ at an overpotential of 0.5 V) and exhibits durability in proton exchange membrane water electrolysers.

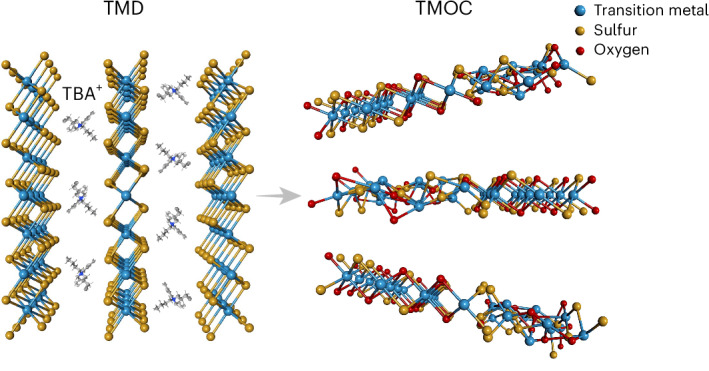

## Abstract (web summary)

A family of two-dimensional transition metal oxychalcogenides is synthesized from bulk transition metal dichalcogenides by tetrabutylammonium intercalation. The stoichiometry and properties of the two-dimensional transition metal oxychalcogenides can be tuned, enabling high stability and catalytic activity for oxygen evolution in acid.

## Main

Liquid-phase exfoliation is a scalable method to produce two-dimensional (2D) nanosheets^1^. There are two approaches for liquid-phase exfoliation of layered transition metal dichalcogenides (TMDs)^2^: one in which the solvent intercalates between the layers of TMDs without reacting to separate them into nanosheets^3^; the other involves a chemical reaction so that 2D nanosheets with properties different from the parent bulk materials are obtained^4^. For example, exfoliation of layered TMDs such as MoS_2_ using butyllithium chemistry leads to transformation from the semiconducting to the metallic phase that is useful for applications in catalysis and energy storage^5–8^. By contrast, electrochemical exfoliation of TMDs using molecular ion intercalation has been used to synthesize large quantities of high-quality TMD nanosheets for high-performance electronics and opto-electronic devices^9^. Compared with TMDs, most transition metal oxides (TMOs) are non-layered^10,11^. Some TMOs are also electrically insulating and thus cannot act as active components in devices or catalysis. However, TMOs are typically more stable than TMDs, which makes them useful for applications where materials are exposed to harsh environments^12^. An example of this is the water oxidation reaction in acidic environments. Proton exchange membrane (PEM) electrolysis allows efficient and sustainable generation of hydrogen energy^13^, but the rare-metal-containing oxygen evolution reaction (OER) catalysts have high cost, leading to serious obstacles^14–17^. Earth-abundant materials such as TMDs that consist of catalytically active transition metals cannot be used for OER because of their poor stability in acids^18^. By contrast, TMOs such as HfO_2_ are stable in acids, but their poor electrical conductivity limits catalytic performance^19,20^.

Transition metal oxychalcogenide (TMOC) materials reported thus far in the literature consist of transition metals such as cobalt (group IX) and bismuth (group XV)^21,22^ or alkali (group I)^21,23^, alkaline earth (group II) and rare earth metals^24^. These TMOCs have discrete compositions and show crystalline structures. They are known for their physical properties that enable them to be useful in various types of device^22,24–27^. In this Article, we report a general and scalable method for synthesizing groups IV and V TMOC nanosheets (HfS_*x*_O_*y*_, ZrS_*x*_O_*y*_, TiS_*x*_O_*y*_, TaS_*x*_O_*y*_, NbS_*x*_O_*y*_, VSe_*x*_O_*y*_, HfSe_*x*_O_*y*_, ZrSe_*x*_O_*y*_ and TaTe_*x*_O_*y*_, where *x* and *y* are S and O concentrations, respectively; *x*, *y* = 0–2) by electrochemical exfoliation. They have mixed structures consisting of crystalline regions (mostly TMDs) and amorphous regions (mostly TMOs). By tuning the exfoliation conditions, a full compositional range between TMDs and TMOs is achieved. Our TMOCs possess catalytic properties and exhibit potential in energy conversion applications.

## Results and discussion

### Synthesis of TMOCs

**Fig. 1:**
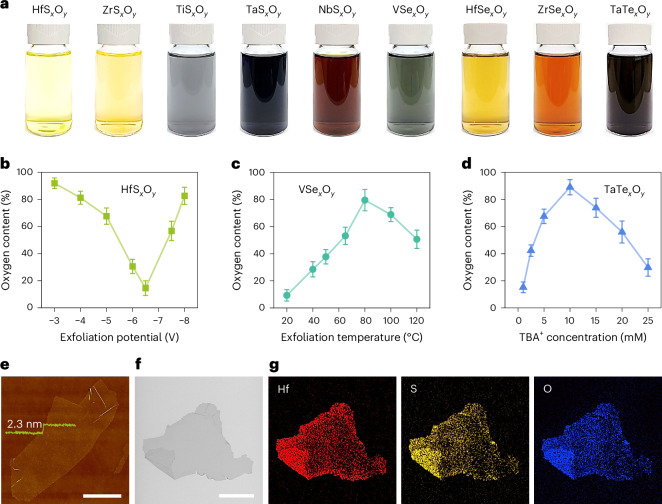
Synthesis of ultrathin TMOCs. **a**, Suspensions of exfoliated TMOC nanosheets. **b**–**d**, Oxygen content [ratio of O to (S + O)] under varying exfoliation conditions. The effects of exfoliation potential on HfS_*x*_O_*y*_ (**b**), exfoliation temperature on VSe_*x*_O_*y*_ (**c**) and TBA^+^ concentration on TaTe_*x*_O_*y*_ (**d**) are presented. The error bars represent the s.d. based on three independent experiments. **e**, Atomic force microscope image of a representative nanosheet with the corresponding height profile. **f**,**g**, Scanning electron microscope image (**f**) and the corresponding EDX elemental maps (**g**) of exfoliated HfS_*x*_O_*y*_. Scale bars, 5 µm. Source data

The electrochemical exfoliation was achieved (Methods and Supplementary Fig. 1) by cathodic intercalation of molecular ions of tetrabutylammonium (TBA^+^) between the layers of TMD bulk crystals. This results in volume expansion and exfoliation of nanosheets (Fig. 1a). The exfoliation parameters such as applied potential, electrolyte temperature and TBA^+^ concentration (Fig. 1b–d) affect intercalation strength, intercalation efficiency and defect formation. The nanosheets are exfoliated down to a few layers with a typical thickness being ~2.3 nm (Fig. 1e). Mild exfoliation with short duration (moderate potential, moderate TBA^+^ concentration, room temperature) gives rise to a low density of chalcogen vacancies, resulting in MX_2‒*x*_^2^, where M is transition metal and X is chalcogen. A high degree of exfoliation with high potential, long duration or elevated temperature helps to generate a high density of chalcogen vacancies and more disordered structures. We found that the TMD oxidises at normal pressure (1 atm) to form TMOC nanosheets (Fig. 1f) with the stoichiometry of MX_*x*_O_*y*_, as suggested by chemical analysis using electron dispersive X-ray spectroscopy (EDX; Fig. 1g). The oxygen content can be tuned by modifying the exfoliation conditions as shown in Fig. 1b–d.

### Atomic structure and chemistry

**Fig. 2:**
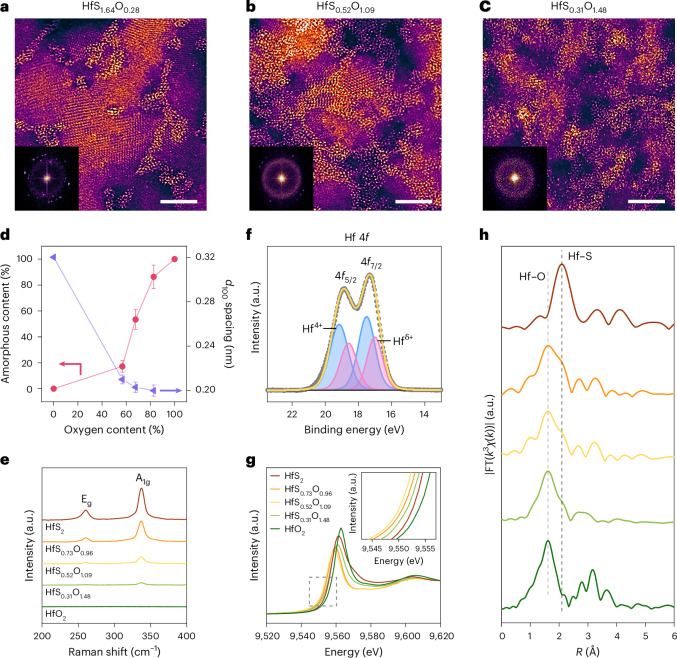
Structural evolution of HfS_*x*_O_*y*_ with varying oxygen content. **a**–**c**, Crystalline/amorphous HfS_1.64_O_0.28_ (**a**), HfS_0.52_O_1.09_ (**b**) and HfS_0.31_O_1.48_ (**c**). Scale bars, 5 nm. The diffraction spots and the diffraction rings in the FFT patterns correspond to the crystalline and the amorphous structures, respectively. The multiple sets of diffraction points in **a** show the various lattice directions due to grain rotations and indicate the polycrystalline nature of the nanosheet^53,54^. Images in **a**–**c** were obtained by HAADF-STEM. Insets: FFT diffraction patterns obtained from the images. **d**, Amorphous content [a real ratio of amorphous regions to (amorphous + crystalline) regions] and *d*_100_ spacing as a function of oxygen content in HfS_*x*_O_*y*_. Error bars represent the s.d. of three independent imaging results. **e**, Raman spectra of HfS_*x*_O_*y*_ with HfS_2_ and HfO_2_ for comparison. **f**, Hf 4*f* core-level XPS spectrum of HfS_0.52_O_1.09_. The shaded peaks in **f** represent contributions from the two oxidation states (4+ and 5+), as indicated. **g**, Hf L_3_-edge XANES spectra. Inset: an enlarged view of the grey box, where the edge position shifts show the different valence states. **h**, Hf L_3_-edge EXAFS spectra of HfS_2_ and HfS_*x*_O_*y*_ with increasing oxygen content and HfO_2_. The vertical lines in **h** represent the peaks from Hf–O and Hf–S bonds. Source data

The atomic structure of the exfoliated TMOCs at different oxygen concentrations was examined by high-angle annular dark-field scanning transmission electron microscopy (HAADF-STEM). The TMOCs are stable under STEM observation (Supplementary Fig. 2). The HfS_*x*_O_*y*_ shows a hybrid structure consisting of crystalline and amorphous regions as shown in Fig. 2a–c. By contrast, HfS_2_ is crystalline (Supplementary Fig. 3a) and HfO_2_ is amorphous (Supplementary Fig. 3b). The mixed crystalline/amorphous structures have also been observed for other TMOCs (Supplementary Fig. 4). The polymorphs of the crystalline regions of exfoliated TMOCs are the same as those of their parent TMDs that we obtained by commercial routes (Supplementary Figs. 3a, 4 and 5a). To further investigate the structure of TMOCs, we combined HAADF-STEM with EDX. As shown in Supplementary Fig. 5b–d, the crystalline region is primarily composed of TMDs and the amorphous region is primarily composed of TMOs. Some O atoms are also present in the crystalline regions and S atoms are also present in the amorphous regions. The O atoms in the crystalline regions are incorporated substitutionally (Supplementary Fig. 5a). With increasing oxygen content, the fraction of amorphous regions increases and the *d*_100_ spacing acquired from fast Fourier transform (FFT) patterns decreases (Fig. 2d). The lattice shrinkage is attributed to the formation of defects such as S vacancies^28^. The vibrational properties of the TMOCs were measured by Raman spectroscopy. It can be seen from Fig. 2e that the characteristic E_g_ and A_1g_ vibrational modes of HfS_2_ are suppressed with increasing oxidation^29^. Similar trends were observed in the Raman spectra for other TMDs. For example, the integrated intensities of characteristic peaks of NbS_2_ and VSe_2_ decrease as a function of oxygen content (Supplementary Fig. 6). The *E* modes disappear at high oxygen content, signifying the breakage of in-plane Nb−S and V−Se bonds^30,31^. As VSe_2_ is oxidized, a peak corresponding to elemental Se is observed^32^. This is due to leaching of Se atoms from the VSe_2_ lattice, which can induce amorphization^31,33^.

The chemical states and coordination environment of the transition metals in TMOCs were examined using X-ray photoelectron spectroscopy (XPS) and X-ray absorption fine structure (XAFS) spectroscopy. For group IV TMOCs, the core-level XPS spectra plotted in Fig. 2f and Supplementary Fig. 7a,b show that the transition metals (Hf, Zr, Ti) reside in the +4 state from their sulfides or oxides (for example HfS_2_ or HfO_2_) where they are fully coordinated. The peaks are shifted with oxygen content (Supplementary Fig. 8a), resulting from the different chemical environments of the transition metal that is bound to S or O. The transition metals also reside in +δ states (2 ≤ δ < 4) when they are uncoordinated due to the presence of defects such as chalcogen vacancies^33^. X-ray absorption near edge structure (XANES) spectra of the Hf L_3_ edge in Fig. 2g show that the white-line intensity and absorption energy of HfS_*x*_O_*y*_ are lower compared with those of HfS_2_ and HfO_2_ (lowest for HfS_0.52_O_1.09_), suggesting a decreased oxidation state for Hf (refs. ^14,34^), which is in agreement with the XPS results (Fig. 2f). The bump feature observed in crystalline HfS_2_ is absent in HfS_*x*_O_*y*_ (Supplementary Fig. 9), indicative of a high concentration of defects in HfS_*x*_O_*y*_ (ref. ^35^). According to the HAADF-STEM, XPS and XANES results, the structure of HfS_*x*_O_*y*_ can be understood as HfS_2_ with varying concentrations of S vacancies and O substitutions, rather than as a mixture of pristine TMDs and pristine TMOs. The extended X-ray absorption fine structure (EXAFS) results in Fig. 2h show that HfS_2_ contains mostly Hf−S bonds (black dashed vertical line) and HfO_2_ contains mostly Hf−O bonds (grey dashed vertical line). By contrast, both Hf−O and Hf−S bonds are observed for HfS_*x*_O_*y*_ (ref. ^36^), demonstrating that Hf atoms are chemically coordinated by S and O atoms. The stoichiometry of TMOCs was obtained by fitting the XPS spectra to gain the average oxidation state and the EXAFS spectra to obtain coordination numbers^14,16,34,36^ (see Methods for details and Supplementary Tables 1–4). The XPS spectra of group V TMOCs are presented in Supplementary Figs. 7c,d and 8b. The transition metals (V, Nb and Ta) reside not only in +4 states from their dichalcogenides (for example NbS_2_) and +2 states from their uncoordinated species but also in +5 states from their oxides (for example Nb_2_O_5_). The increase in state from +4 to +5 indicates that oxidation has occurred on the starting TMDs. In contrast to Hf^4+^, the peaks corresponding to Nb^4+^ show negligible shifts regardless of the oxygen content (Supplementary Fig. 8b), suggesting that the chemical state of Nb is increased once oxidized and Nb−O bonds are formed.

### Bandgap and electrical conductivity

**Fig. 3:**
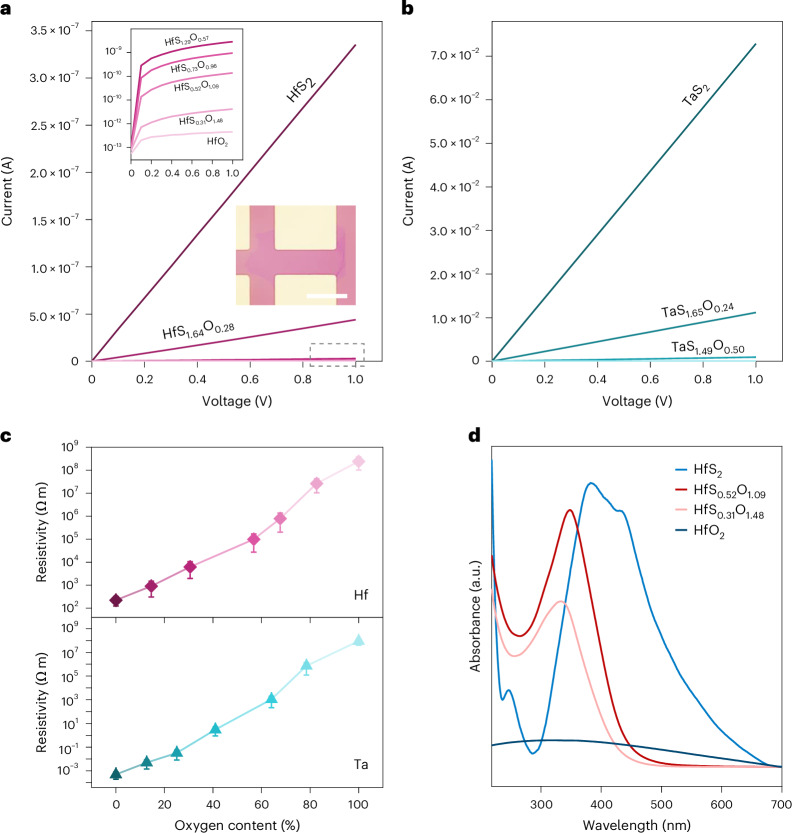
Electrical and optical properties of TMOCs. **a**,**b**, *I*−*V* curves acquired from electronic devices (lower inset; scale bar, 20 µm) where HfS_*x*_O_*y*_ (**a**) and TaS_*x*_O_*y*_ (**b**) act as the channel material. The upper inset in **a** is the logarithmic plots of the curves in the grey box. **c**, Electrical resistivity of HfS_*x*_O_*y*_ and TaS_*x*_O_*y*_ as a function of oxygen content. The results corresponding to pure sulfides and oxides with different oxygen concentrations are shown in **a**–**c** for comparison. Error bars represent the s.d. obtained from three independent devices. **d**, Ultraviolet–visible spectra of HfS_*x*_O_*y*_ with HfS_2_ and HfO_2_ for comparison. Source data

To explore the physical properties, we fabricated devices based on ultrathin TMOCs with increasing oxygen content. The electrical measurements (Fig. 3a,b) demonstrate that the resistivity of TMOCs exfoliated from semiconducting HfS_2_ and metallic TaS_2_ can be tuned by more than six to eight orders of magnitude (Fig. 3c). The optical absorption of TMOCs is shown in Fig. 3d. The corresponding Tauc plots (Supplementary Fig. 10) indicate that the bandgap widens as the oxygen content increases (HfS_2_: ~1.7 eV < HfS_*x*_O_*y*_: ~2.5−2.8 eV < HfO_2_: ~5.0 eV)^37,38^, leading to a decrease in electrical conductivity.

### Electrocatalysis for OER in acid

**Fig. 4:**
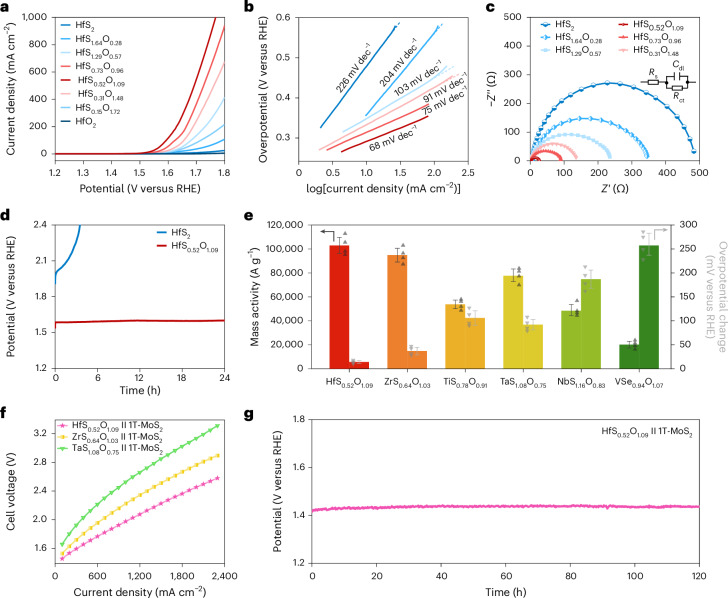
OER catalysis in electrolyte with pH ≈ 0. **a**, Polarization curves for HfS_2_ and HfS_*x*_O_*y*_ with varying oxygen content and HfO_2_ after iR correction. **b**, Tafel slopes acquired from **a**. **c**, Nyquist plots obtained by electrochemical impedance spectroscopy. Inset: the equivalent circuit used to obtain impedance. **d**, Chronopotentiometric tests for HfS_2_ and HfS_0.52_O_1.09_. **e**, Comparison among different TMOCs of mass activity (left) at the overpotential of 0.5 V versus RHE and potential change (right) after 24 h at 50 mA cm^−2^. Error bars represent the s.d. of four independent measurements and the data distribution is shown using grey triangles. **f**, *I*−*V* curves from PEM electrolysers with HfS_0.52_O_1.09_, TaS_1.08_O_0.75_ or ZrS_0.64_O_1.03_ as OER catalyst on the anode and 1T-MoS_2_ as HER catalyst on the cathode. **g**, Stability measurement for HfS_0.52_O_1.09_||1T-MoS_2_ electrolyser operating at a current density of 20 mA cm^−2^. Source data

Based on the above findings, we sought to translate the fundamental material characteristics into proof-of-concept demonstration. We chose OER to investigate the electrochemical activity and stability of TMOCs in acidic media (Supplementary Fig. 11a). Groups IV and V TMDs are not stable as catalysts for OER in acid and their full coordination makes them unfavourable for adsorption of intermediate species^39^. Groups IV and V TMOs can be stable at low pH but are too insulating for the reaction to proceed. Therefore, groups IV and V TMOCs with uncoordinated transition metal atoms in their structure may be suitable electrocatalysts. To validate this, we have studied the OER electrocatalysis of TMOC nanosheets at pH = 0.25. Figure 4a shows the polarization curves [current density as a function of applied potential (versus a reversible hydrogen electrode, RHE)] of HfS_*x*_O_*y*_ nanosheets with different stoichiometries, along with HfS_2_ and HfO_2_ for comparison. The HfS_0.52_O_1.09_ exhibits a current density of >1,000 mA cm^−2^ at a potential of <1.8 V versus RHE. The polarization curves of some other TMOCs are shown in Supplementary Fig. 12. The potentials have been iR-corrected (where iR indicates voltage loss (that is, iR drop) due to internal resistance of measurement set-up; R is resistance of the electrolyte solution) and normalized to the electrochemical surface area obtained by cyclic voltammetry (CV) at different scan rates (Supplementary Fig. 13). The highest double-layer capacitance (*C*_dl_) for HfS_0.52_O_1.09_ indicates that it has the largest density of active sites compared with other Hf-based catalysts. The average overpotential for HfS_0.52_O_1.09_ was found to be 295 ± 7 mV versus RHE at a current density of 10 mA cm^−2^ and the average Tafel slope was 64 ± 5 mV dec^−1^ (Supplementary Fig. 14). The Nyquist plots in Fig. 4c indicate the lower electrochemical impedance of HfS_0.52_O_1.09_—facilitating the charge transfer and allowing the OER to proceed more efficiently.

The stability of the TMOCs at pH = 0.25 was monitored by recording the potential (versus RHE) required to maintain a current density of 50 mA cm^−2^ with time. The overpotential only shows a small increase (14 ± 3 mV for HfS_0.52_O_1.09_, 37 ± 8 mV for ZrS_0.64_O_1.03_ and 92 ± 10 mV for TaS_1.08_O_0.75_) after 24 hours and the TMOCs are more durable than TMDs (Fig. 4d and Supplementary Fig. 16). This originates from the fact that the S^2−^ in TMOCs is stabilized by hybridization of the S 3*p* and O 2*p* orbitals. Hence, they are more robust against self-oxidation^40^. TMOCs were characterized by XPS after stability tests and a slight increase in the chemical states was observed for Hf, suggesting good stability in acid electrolyte, whereas those for Ta were increased to a larger extent (Supplementary Fig. 17).

Mass activity indicates how much current is produced by loading per unit amount of a catalyst. Low-dimensional catalysts are desired for high mass activity owing to their high specific surface area and enriched active sites^41^. The average mass activity of HfS_0.52_O_1.09_ for OER in acidic media (pH = 0.25) is 102,959 ± 6,834 A g^−1^ at an overpotential of 0.5 V versus RHE. The exfoliation conditions were adjusted to obtain TMOCs showing the highest activity (Supplementary Table 5). The mass activities of the optimized TMOCs are compared in Fig. 4e. While there is lack of reference data for earth-abundant ultrathin OER catalysts in acid^41^, the mass activity of HfS_0.52_O_1.09_ is higher than those of many other catalysts that are measured in alkaline media (pH = 13 or 14; Supplementary Fig. 18a and Supplementary Table 6). Moreover, the stability is also compared in terms of overpotential change after 24 hours at 50 mA cm^−2^ (Fig. 4e). The stability of HfS_0.52_O_1.09_ is comparable to those reported for similar materials^39,42–46^ (Supplementary Fig. 18b and Supplementary Table 7). The activities of the TMOC catalysts that we have optimized for OER at pH ≈ 0 are in the order: HfS_0.52_O_1.09_ > ZrS_0.64_O_1.03_ > TaS_1.08_O_0.75_ > TiS_0.78_O_0.91_ > NbS_1.16_O_0.83_ > VSe_0.94_O_1.07_. The activity–stability balance of HfS_0.52_O_1.09_ demonstrates that it is viable for realistic applications.

To further evaluate the potential of TMOCs for producing hydrogen, we constructed PEM water electrolysers with treated Nafion 117 membrane^14,16^ (Supplementary Fig. 11b). This is a state-of-the-art PEM electrolyser where the catalysts are free from rare earth or noble metal elements^14–16,33,47^. TMOC (HfS_0.52_O_1.09_, ZrS_0.64_O_1.03_ or TaS_1.08_O_0.75_) nanosheets were employed as the anode for OER and 2D 1T-MoS_2_ as the cathode for hydrogen evolution reaction (HER). The characterization and catalytic activities of 1T-MoS_2_ are shown in Supplementary Fig. 19. The electrolysers suggest that high current densities (Fig. 4f) and operation for 120 hours (Fig. 4g and Supplementary Fig. 20) can be achieved with TMOCs.

In summary, we have synthesized a family of ultrathin materials, namely, TMOC nanosheets. The control of synthesis enables us to tune the composition and structure of TMOCs. TMOCs have unique properties compared with TMDs and TMOs, which may render them promising in applications that are unlikely to be realized by TMDs or TMOs. The synthesis of TMOCs provides the community an open strategy of material design for targeted functionalities.

## Methods

### Materials synthesis

The TMOC nanosheets were synthesized by electrochemical exfoliation using a potentiostat (ModuLab XM, Solartron Analytical, Ametek Scientific Instruments or CHI 760E, Shanghai CH Instrument). Bulk crystals of TMDs (2D Semiconductors or HQ Graphene) acted as the cathode, a carbon rod acted as the anode and the electrolyte was tetrabutylammonium tetrafluoroborate (TBATFB, 99%, Sigma Aldrich) dissolved in *N*,*N*-dimethylformamide (DMF, 99.8%, Thermo Fisher Scientific). For each batch, the size of the TMD crystal was ~0.01 mm^2^ and the anode-to-cathode distance was fixed at 1.2 cm. When a negative potential was applied, the TBA^+^ cations along with the DMF molecules co-intercalated into these layered materials, so that cathodic reduction and exfoliation took place simultaneously^2^. The exfoliation was controlled by tuning the cathodic potential, TBA^+^ concentration and electrolyte temperature, the parameters of which were kept constant during the whole exfoliation. The potential was applied by chronoamperometry. A thermocouple was immerged into the electrolyte to monitor the temperature. For comparison, single-crystalline TMD flakes were prepared by mechanical exfoliation with Scotch tapes. The 1T-MoS_2_ was synthesized by chemical exfoliation using *n*-butyllithium solution in hexane (1.6 M, Sigma Aldrich) as the intercalation agent, the protocol of which was elaborated previously^5^. To rinse the electrochemically exfoliated products, the suspension was rested until the nanosheets completely sedimented. Then, the TBATFB‒DMF electrolyte was removed and ethanol (≥99.8%, Thermo Fisher Scientific) was poured in, followed by gentle shaking of the vial to disperse the nanosheets. The above steps were repeated several times. Then, the samples were drop-casted onto a substrate and dried under normal temperature and pressure (20 °C and 1 atm) before characterization and measurements.

### Characterization

The nanosheets were supported by gold (100 nm)-coated Si chips for scanning electron microscopy, EDX and XPS, on SiO_2_ (300 nm)/Si chips for atomic force microscopy and Raman spectroscopy and on copper grids covered with carbon films for HAADF-STEM. The imaging took place at room temperature utilizing a scanning electron microscope (Nova NanoSEM 450, FEI) equipped with an energy-dispersive detector at an accelerating voltage of 15.0 kV and a high-angle annular dark-field scanning transmission electron microscope (JEM-ARM200F, JEOL) equipped with double hexapole C_S_ correctors (CEOS GmbH) at an accelerating voltage of 200 kV. The samples were stored in ultrahigh vacuum overnight before EDX mapping to minimize the physiosorbed O species. The nanosheet thickness was examined using an atomic force microscope (Bruker Dimension FastScan) in tapping mode. The Raman spectra were recorded on a confocal Raman microscope (alpha300 R, WITec). A 1 mW, 532 nm (2.33 eV) diode laser was employed for excitation (spot size of 1 μm, acquisition time of 10 s). XPS was conducted using a spectrometer (ESCALab250Xi, Thermo Scientific) in ultrahigh vacuum (base pressure of 3 × 10^−10^ mbar) with X-ray Al Kα monochromatic light (energy of 1,486.84 eV). The diameter of the circular light spot was adjustable in the range 900–100 μm. The testing depth was 3–7 nm. The XANES and EXAFS spectra were recorded at the Singapore Synchrotron Light Source using a Si(111) double-crystal monochromator at an accelerating voltage of 700 MeV and a beam current of 200 mA. The stoichiometry of TMOCs was determined by XPS (and EXAFS) measurements. For each TMOC, the core-level XPS spectrum of the transition metal was fitted to obtain its chemical states. The stoichiometric ratio of transition metal to (S + O) was estimated by integrating the areas under the curves for each state and calculating the percentage of each state. For Hf-based TMOCs, the EXAFS spectra were fitted to estimate the ratio of S to O, considering the small difference in binding energy between Hf–S and Hf–O (Fig. 2f). For other TMOCs, we used XPS to suggest the atomic ratio between chalcogen and oxygen, then high-resolution O 1*s* XPS spectra were fitted to obtain the percentage of chemically bonded oxygen by excluding the contributions from chemisorbed and physisorbed oxygen^14,48^ (see Supplementary Fig. 7c as an example).

### Electrochemical measurements

The catalytic performance was measured in N_2_-purged aqueous solutions of 0.5 M H_2_SO_4_ (Sigma Aldrich). The pH value was 0.25, as determined by a microprocessor pH meter (Hanna Instruments). A three-electrode system was employed (ModuLab XM, Solartron Analytical, Ametek Scientific Instruments or CHI 760E, Shanghai CH Instrument). The working electrode was fabricated as below. The catalysts were rinsed using ethanol (≥99.8%, Thermo Fisher Scientific), after which 20 µl Nafion 117 solution (5 wt%, Sigma Aldrich) was added into 1 ml of the suspension. Once dispersed, the mixture was drop-cast onto a glassy carbon surface and dried in vacuum. Other supports including carbon paper (190 µm, polytetrafluoroethylene-treated for 5% wet proofing, Toray Industries) and fluorine-doped tin oxide (FTO)-coated glass (750 nm/1 mm, Ossila) were also used to evaluate the reproducibility of the results. The mass loading of the catalysts was 0.1 mg. A carbon rod and standard calomel electrode (SCE) were chosen as the counter and reference electrodes, respectively. The polarization curves were obtained by linear sweep voltammetry (LSV) under quasi-equilibrium conditions at a scan rate (*k*_s_) of 5 mV s^−1^. All the potentials measured against SCE were calibrated to RHE using the equation: *E*(RHE) = *E*(SCE) + 0.241 V + 0.0591 × pH and were corrected by iR compensation. The electrolyte resistance was determined as the high-frequency intercept of the Nyquist plot acquired from electrochemical impedance spectroscopy (EIS). The electrochemical surface area is the ratio of *C*_dl_ to *C*_s_ ($\frac{{C}_{\mathrm {dl}}}{{C}_{\mathrm {s}}}$, where *C*_s_ represents the specific capacitance). CV was carried out at different *k*_s_ (5−120 mV s^−1^), then *C*_dl_ was estimated by deriving the slope value from the linear regression between half of the current density variation and the scan rate ($\frac{\varDelta {j}_{\mathrm {geo}}}{2}$ versus *k*_s_). *C*_s_ is expressed as $\frac{{A}_{\mathrm {CV}}}{2\times {k}_{\mathrm {s}}\times \varDelta V\times {A}_{\mathrm {geo}}}$, where *A*_CV_ is the area of the CV curve at a certain *k*_s_, Δ*V* is the potential window and *A*_geo_ is the geometric area of catalysts on the substrate. In PEM water electrolysis, the anode and the cathode were prepared by depositing the OER catalyst (TMOCs) and the HER catalyst (2D 1T-MoS_2_), respectively, onto carbon paper. The cell was separated by a perfluorinated Nafion membrane (Nafion 117, Sigma Aldrich). The surface area of the electrodes was 1 cm × 1 cm. The polarization curves of the PEM water electrolysers were recorded from the current density of 100 to 2,300 mA cm^−2^. The stability of OER and PEM water electrolysis was investigated by measuring the potential versus RHE and the cell voltage, respectively, while keeping the current density at constant values. The cell voltages of the water electrolysers were not iR-corrected. Potential reduction could take place at the beginning of the chronopotentiometric tests, which might be due to the activization of the system.

### Density functional theory calculations

Density functional theory calculations were performed using the plane-wave technique in the Vienna Ab initio Simulation Package (VASP)^49,50^. The projected augmented wave (PAW) pseudo-potentials^51^ and the Perdew–Burke–Ernzerhof (PBE) exchange–correlation functional were adopted in all calculations^52^. The energy cutoff was set as 400 eV for the plane-wave basis set for the sake of high accuracy. A vacuum layer of 20 Å was used to avoid the interference of the adjacent periodic images. For supercells featuring distinct geometries or sizes, Monkhorst−Pack *k*-point sampling was implemented with a grid spacing of ~0.03 Å^−1^ along each reciprocal lattice vector within the Brillouin zone. The energy and force convergence criteria were set to 10^−4^ eV and 0.01 eV Å^−1^, respectively. The computational hydrogen electrode (CHE) method was applied to determine the Gibbs free energy (Δ*G*) of the reaction intermediates (OH*, O* and OOH*), using the following equation

1 $$\Delta G=\Delta E+\Delta {E}_{\mathrm {ZPE}}-T\Delta S$$

where Δ*E*, Δ*E*_ZPE_ and Δ*S* represent the difference between the total energy, zero point energy and entropy, respectively and *T* represents the temperature (298.15 K). The electrochemical potential of an electron–proton pair (H^+^ + e^−^) is equal to the free energy of half a hydrogen molecule (1/2 H_2_) at standard pressure.

## Supplementary information

Supplementary Information Supplementary Figs. 1–21 and Tables 1–7.

## Source data

Source Data Fig. 1 Numerical data used to generate graphs of Fig. 1b−d.

Source Data Fig. 2 Numerical data used to generate graphs of Fig. 2d−h.

Source Data Fig. 3 Numerical data used to generate graphs of Fig. 3.

Source Data Fig. 4 Numerical data used to generate graphs of Fig. 4.

## Data Availability

All data that support the findings of this study are available in the article and its Supplementary Information. Source data are provided with this paper.
