# Supplementary material for: Ultrathin transition metal oxychalcogenide catalysts for oxygen evolution in acidic media
Source: Nat Synth. 2025 Jan 2;4(3):327–35. doi: 10.1038/s44160-024-00694-3 (PMC11903303; doi:10.1038/s44160-024-00694-3)
Supplement: Supplementary file 1 — Supplementary Figs. 1–21 and Tables 1–7. [file 44160_2024_694_MOESM1_ESM.pdf]

nature synthesis

Supplementary information https://doi.org/10.1038/s44160-024-00694-3

Ultrathin transition metal oxychalcogenide catalysts for oxygen evolution in acidic media

In the format provided by the authors and unedited

Table of Contents

Supplementary Fig. 1 Schematics of the electrochemical exfoliation 3

Supplementary Fig. 2 HAADF-STEM imaging with time 4

Supplementary Fig. 3 HAADF-STEM images of HfS_2 and HfO_2 4

Supplementary Fig. 4 HAADF-STEM images of TMOCs 5

Supplementary Fig. 5 HAADF-STEM images and EDX maps of HfS_xO_y 6

Supplementary Fig. 6 Raman spectra of TMOCs and TMDs 7

Supplementary Fig. 7 XPS spectra of TMOCs 8

Supplementary Fig. 8 XPS spectra of Hf- and Nb-based compounds 9

Supplementary Fig. 9 XANES spectra of Hf-based compounds 10

Supplementary Fig. 10 Tauc plots of HfS_xO_y, HfS_2 and HfO_2 10

Supplementary Fig. 11 Schematics of the electrochemical measurements 11

Supplementary Fig. 12 OER catalysis of TMOCs in pH ≈ 0 acid 11

Supplementary Fig. 13 Investigation of C_{dl} 12

Supplementary Fig. 14 Reproducibility of OER measurements 13

Supplementary Fig. 15 DFT calculations 14

Supplementary Fig. 16 Stability measurements of TMOCs and TMDs 14

Supplementary Fig. 17 XPS before and after OER measurements 15

Supplementary Fig. 18 Comparison of OER performances with literature 16

Supplementary Fig. 19 Characterization and measurements of 1T-MoS_2 17

Supplementary Fig. 20 Stability measurements of PEM water electrolysers 18

Supplementary Fig. 21 Exfoliated group 6 TMDs 19

Supplementary Table 1 Fitting parameters for EXAFS of $HfS_{0.73}O_{0.96}$ 20

Supplementary Table 2 Fitting parameters for EXAFS of $HfS_{0.52}O_{1.09}$ 20

Supplementary Table 3 Fitting parameters for EXAFS of $HfS_{0.31}O_{1.48}$ 21

Supplementary Table 4 Determination of the stoichiometric ratio of HfS_xO_y 21

Supplementary Table 5 Synthetic conditions of the optimized TMOC catalysts 21

Supplementary Table 6 Literature survey on mass activity for result comparisons 22

Supplementary Table 7 Literature survey on stability for result comparisons 23

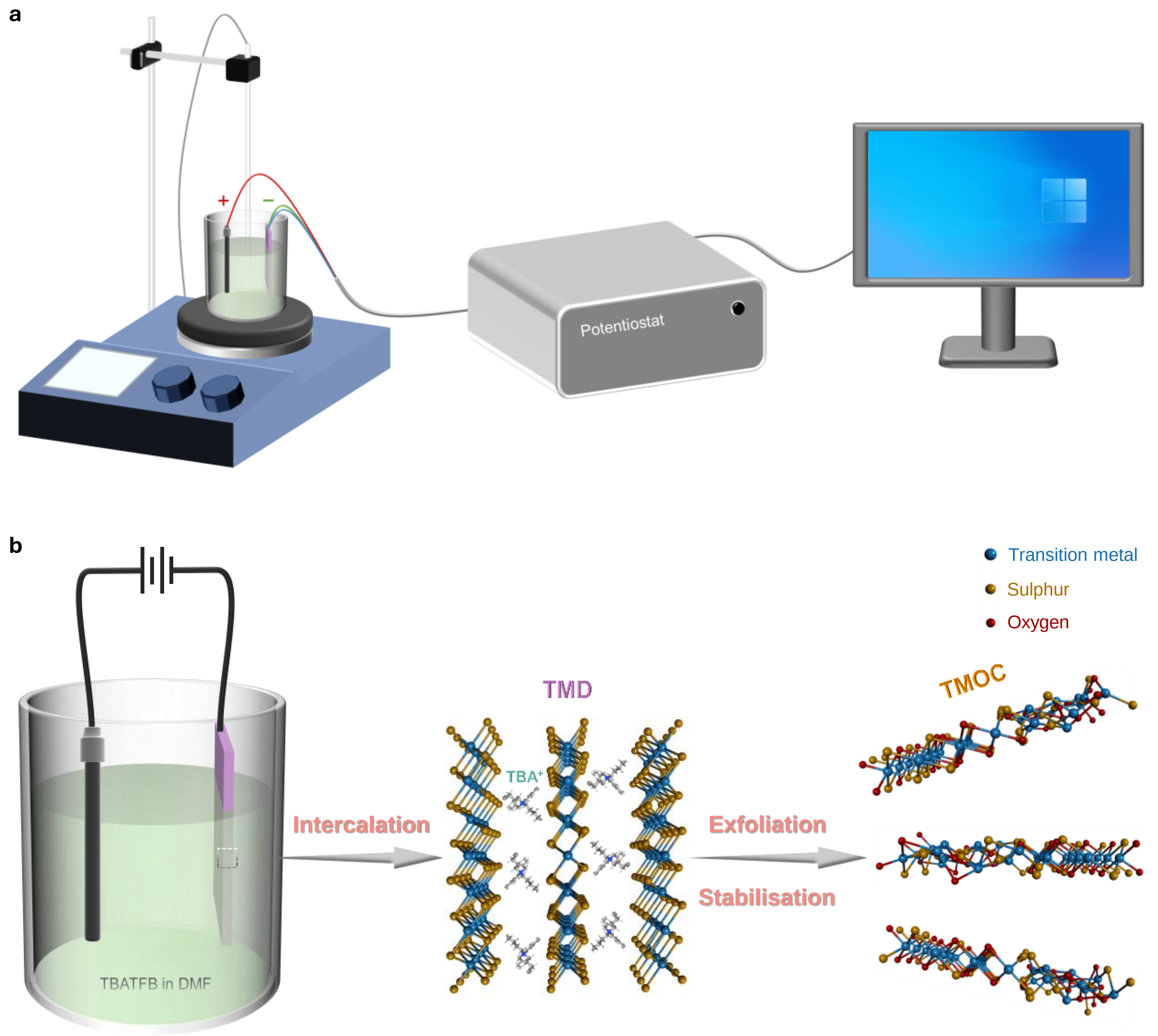

Supplementary Fig. 1 | Schematics of the electrochemical exfoliation. a, Experimental setup. A hot plate with temperature probe, a potentiostat and a computer are used. b, Intercalation and exfoliation processes (see Methods for details). The exfoliated nanosheets contain defects (such as chalcogen vacancies that expose the neighbouring transition metal) and are stabilized via oxidation. Their final structure varies depending on the synthetic conditions, including the applied potential, electrolyte temperature and TBA^+ concentration.

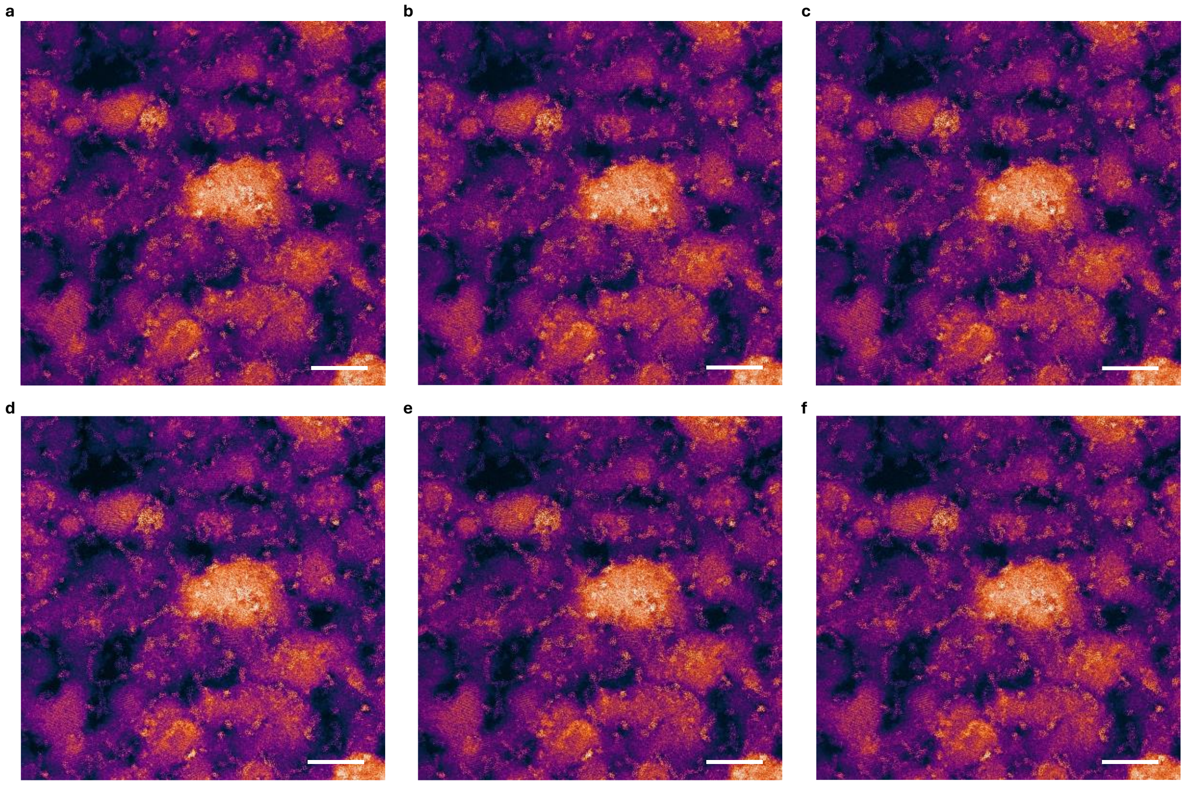

Supplementary Fig. 2 | HAADF-STEM imaging with time. a–f, HAADF-STEM images of $HfS_{1.64}O_{0.28}$ with a time interval of 30 s each. The negligible change excludes the possibility of structural amorphization induced by the electron beam. Scale bars: 10 nm.

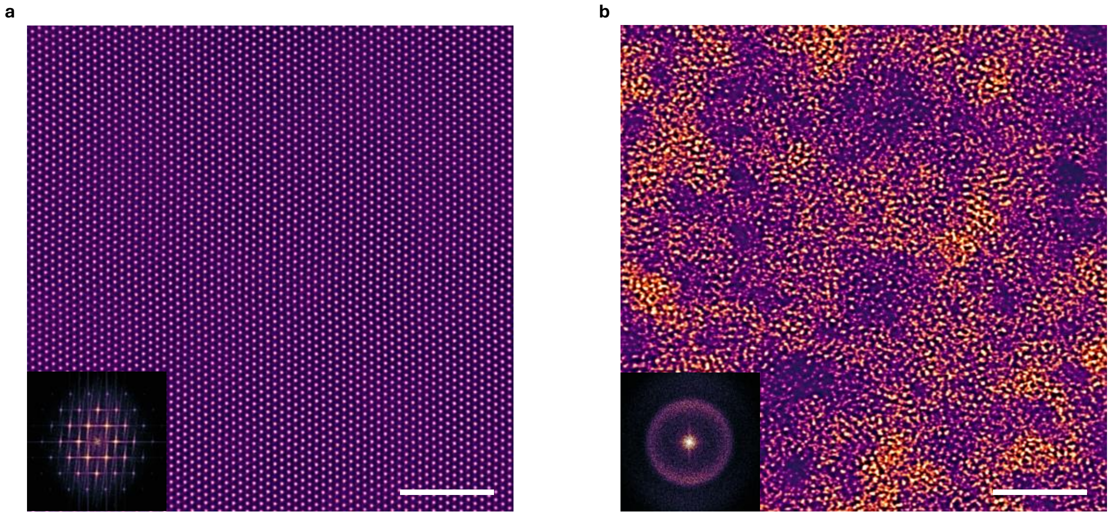

Supplementary Fig. 3 | HAADF-STEM images of HfS_2 and HfO_2. a, Crystalline HfS_2. b,

Amorphous HfO_2. Scale bars: 5 nm. The crystalline and amorphous structures are also manifested by the diffraction spots and diffraction ring in the corresponding FFT patterns (insets), respectively.

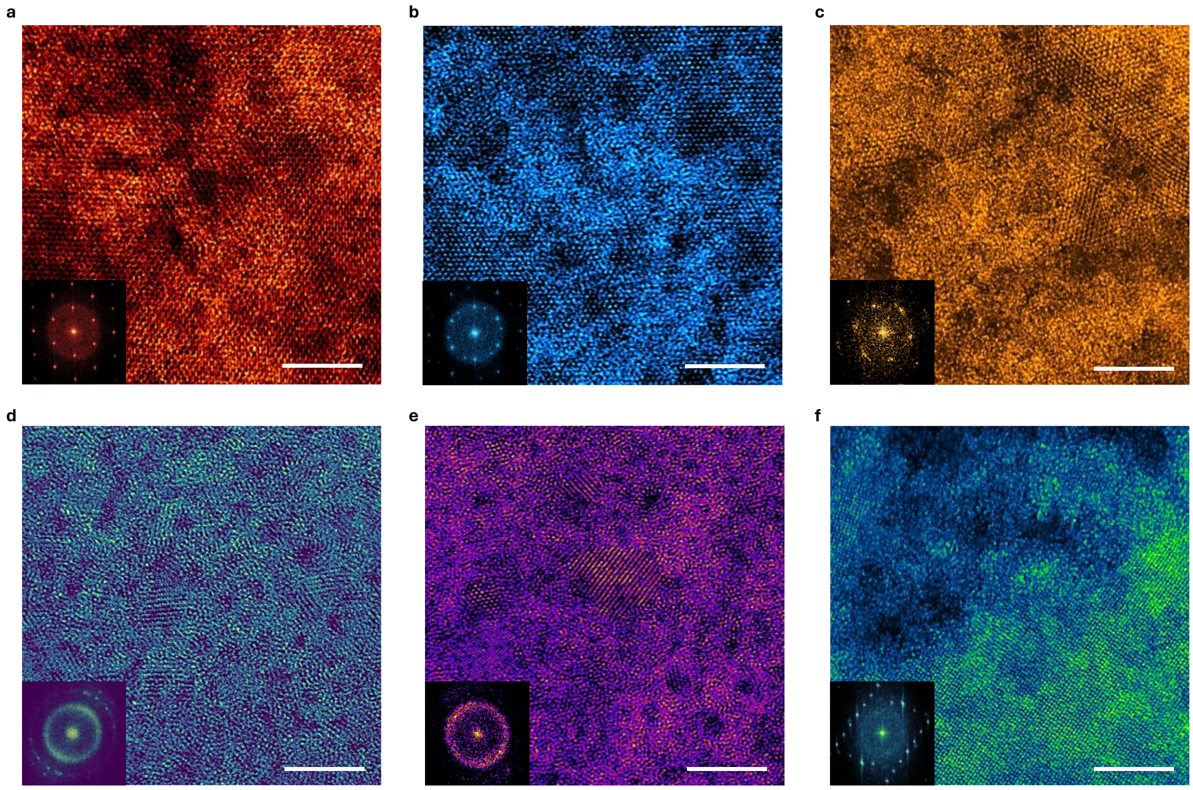

Supplementary Fig. 4 | HAADF-STEM images of TMOCs. a, NbS_xO_y, b, TaS_xO_y, c, VSe_xO_y, d, $ZrSe_xO_y$, e, $HfSe_xO_y$ and f, $TaTe_xO_y$. The corresponding FFT patterns are shown in the insets. Scale bars: 5 nm. These TMOCs consist of a mixture of crystalline and amorphous regions. Layered structures have been observed in (d,e).

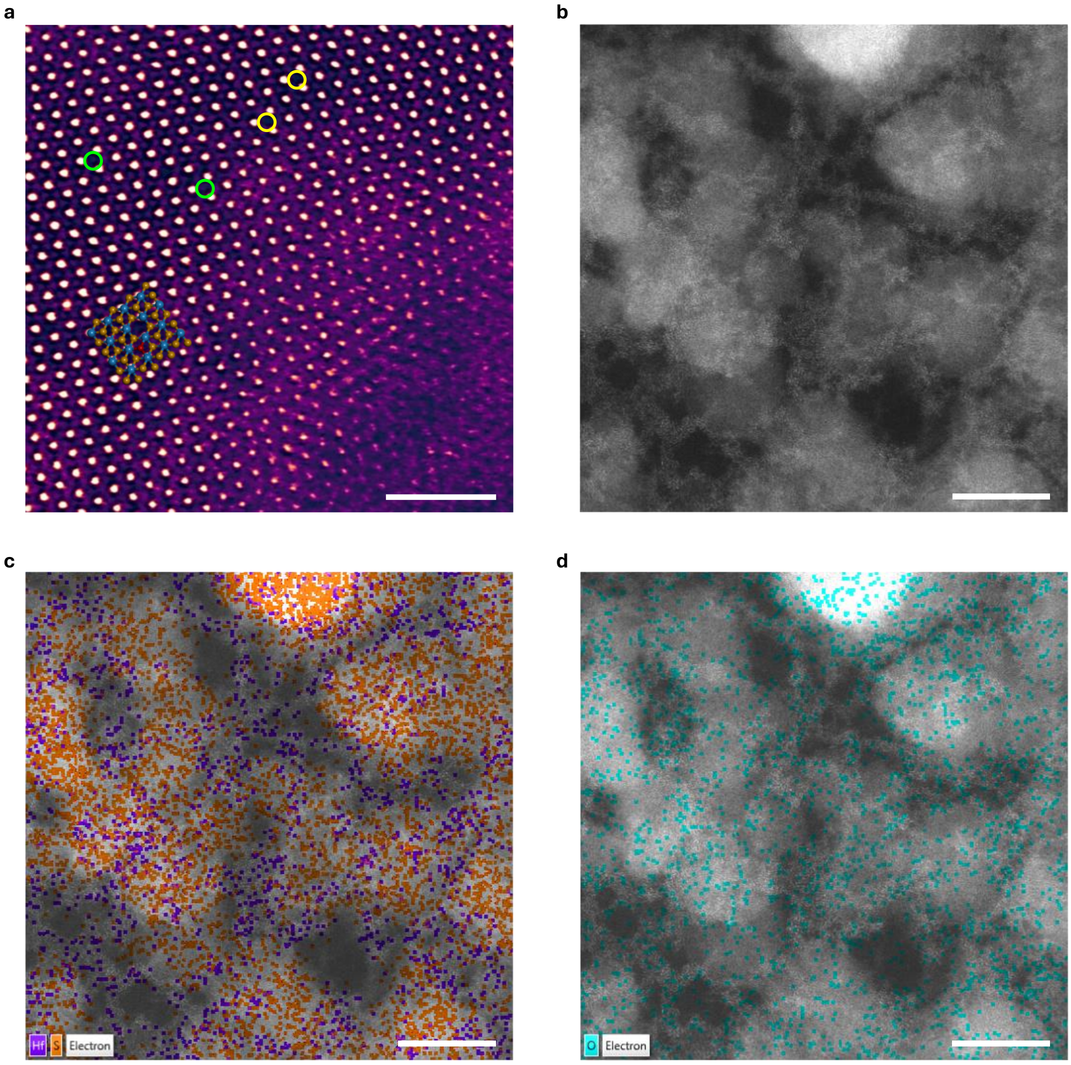

Supplementary Fig. 5 | HAADF-STEM images and EDX maps of HfS_xO_y. a, HAADF-STEM image of $HfS_{1.64}O_{0.28}$. Scale bar: 2 nm. In the crystalline region, S vacancies (yellow circles) and O substitutions (green circles) can be observed, which are also shown by the atomic model (blue balls: Hf, yellow balls: S, red ball: O). The polymorph is identical to that of pristine HfS_2 (Supplementary Fig. 3a). b, HAADF-STEM image of $HfS_{0.52}O_{1.09}$. c,d, Corresponding EDX maps showing the elemental distributions of Hf, S (c) and O (d). Scale bars: 10 nm.

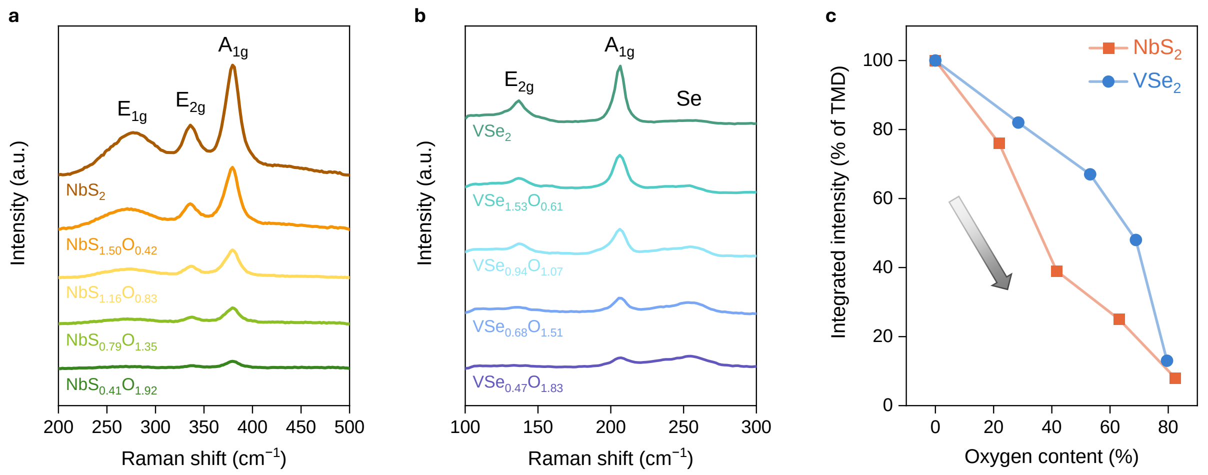

Supplementary Fig. 6 | Raman spectroscopy of TMOCs and TMDs. a, Raman spectra of NbS_2 and NbS_xO_y with increasing oxygen contents. b, Raman spectra of VSe_2 and VSe_xO_y with increasing oxygen contents. c, Integrated intensity of characteristic Raman peaks of NbS_2 and VSe_2 as a function of oxygen content, acquired from (a,b). The integrated intensity of pure NbS_2 and VSe_2 is defined as 100%. The vibrational modes for these TMDs are suppressed with the increase in oxygen content (grey arrow).

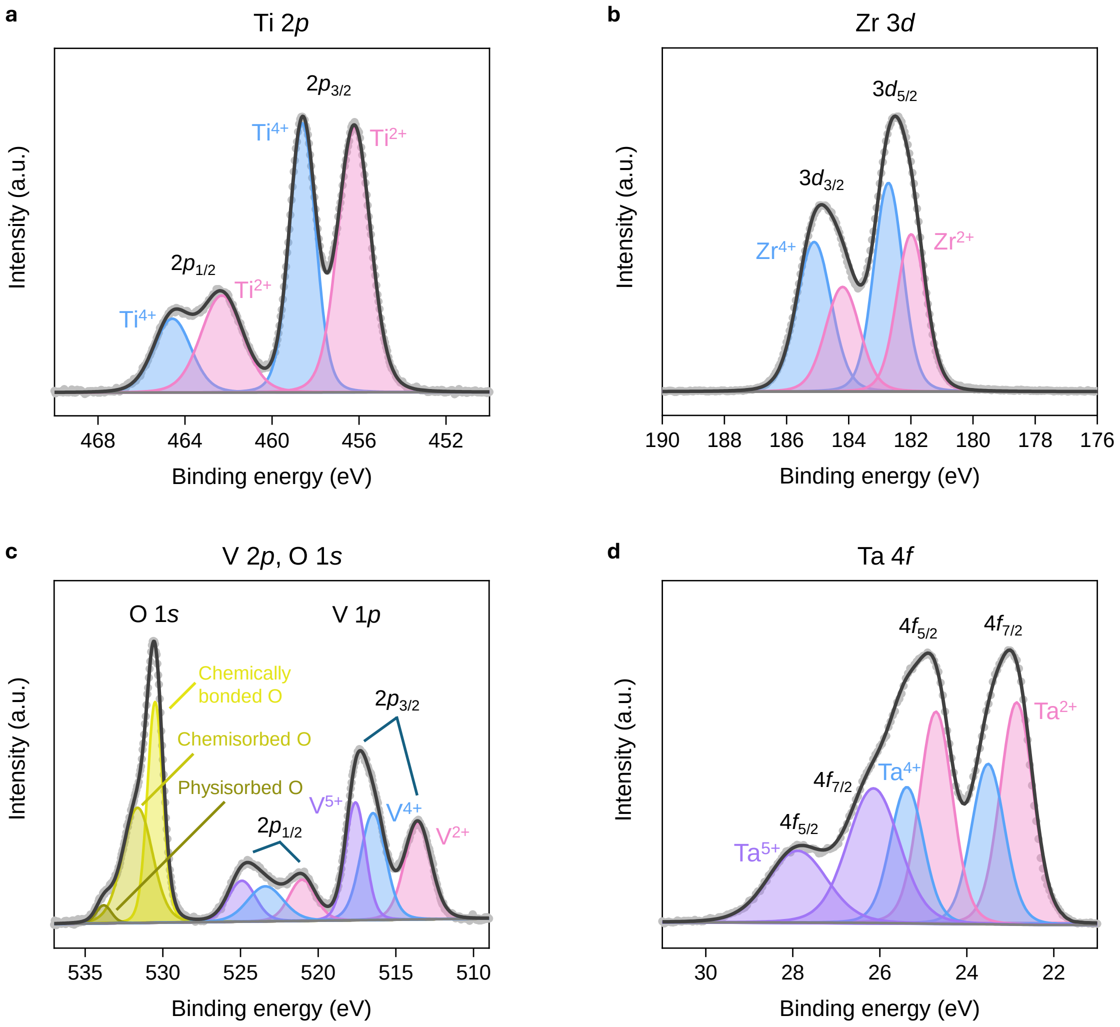

Supplementary Fig. 7 | XPS spectra of TMOCs. a, Ti 2*p* core-level spectrum of TiS_xO_y. b, Zr 3*d* core-level spectrum of ZrS_xO_y. c, V 2*p* and O 1*s* core-level spectra of VSe_xO_y. d, Ta 4*f* core-level spectrum of $TaTe_xO_y$.

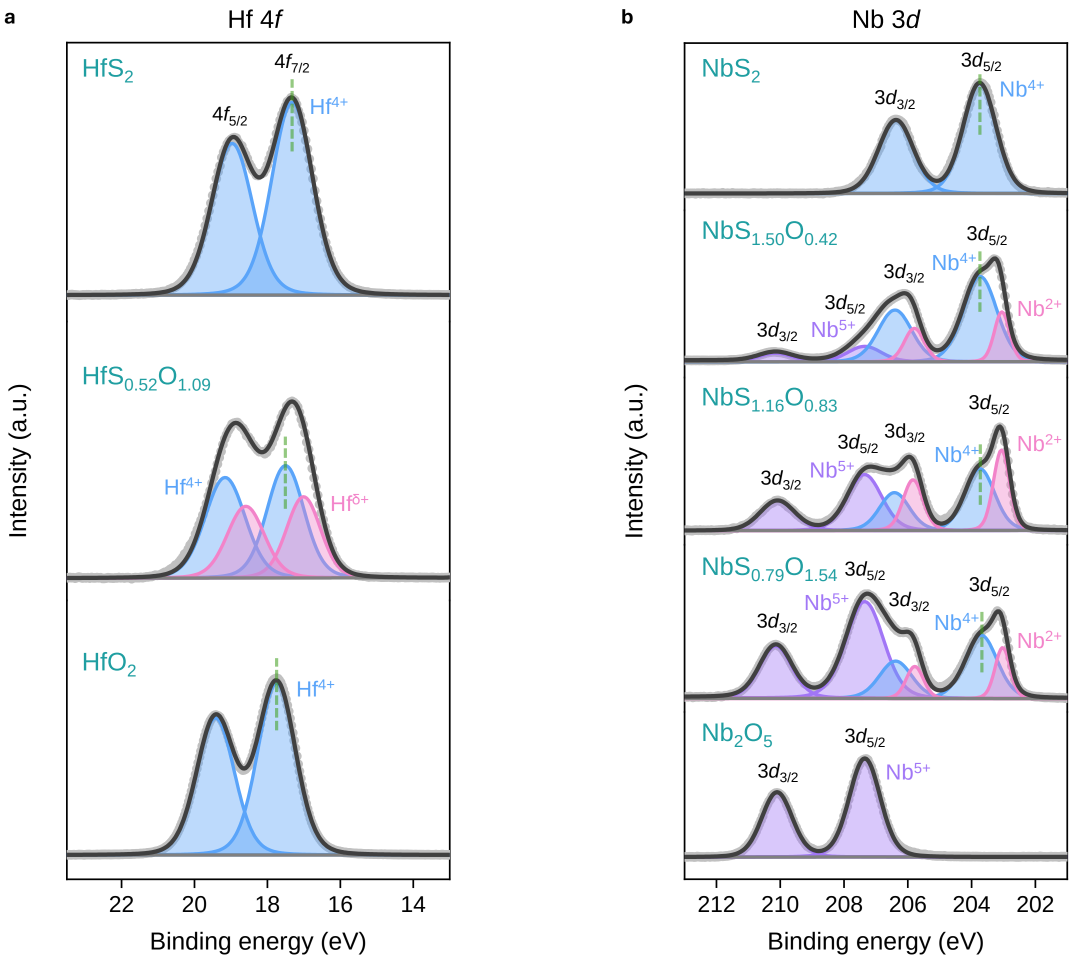

Supplementary Fig. 8 | XPS spectra of Hf- and Nb-based compounds. a, Hf 4*f* core-level XPS spectra of HfS_2, $HfS_{0.52}O_{1.09}$ and HfO_2. b, Nb 3*d* core-level XPS spectra of NbS_2, NbS_xO_y with increasing oxygen contents and Nb_2O_5.

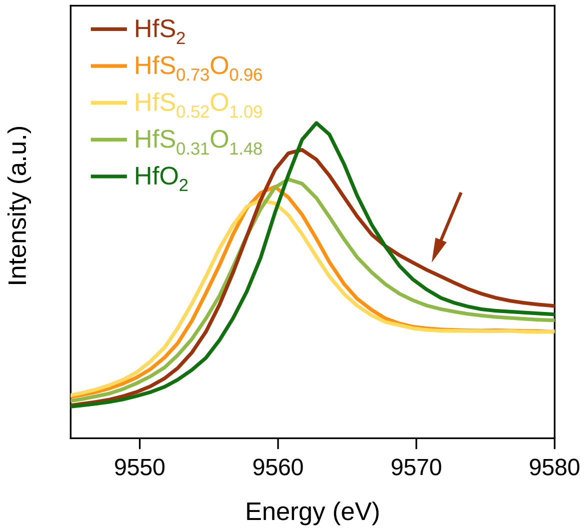

Supplementary Fig. 9 | XANES spectra of Hf-based compounds. The red arrow indicates a bump feature originating from crystalline HfS_2.

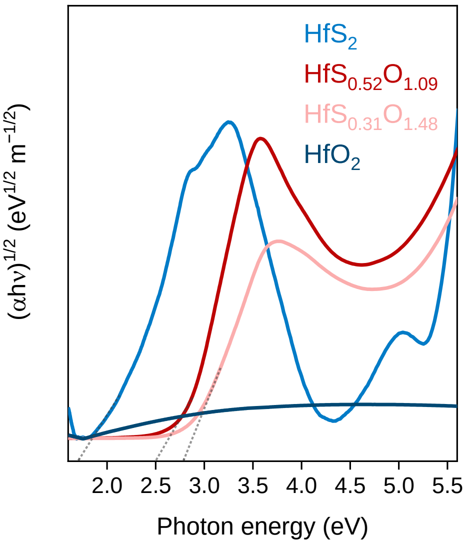

Supplementary Fig. 10 | Tauc plots of HfS_xO_y, HfS_2 and HfO_2. The optical band gaps of HfS_xO_y and HfS_2 were obtained via linear extrapolation of the Tauc plots to the *x*-axis[1], whereas the optical band gap of HfO_2 was estimated from the absorption edge of UV−vis spectrum in Fig. 3d due to the absence of linear region in the Tauc plot[2].

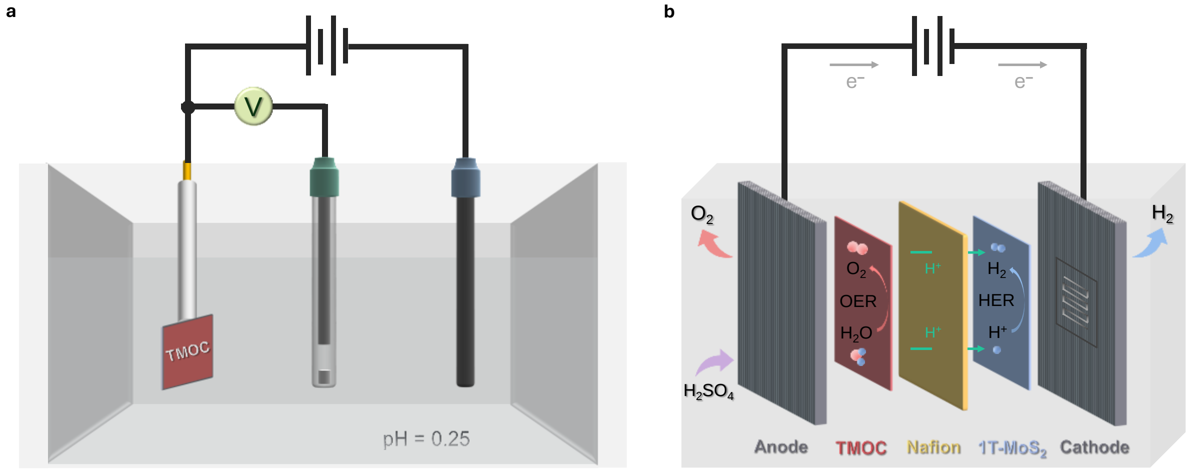

Supplementary Fig. 11 | Schematics of the electrochemical measurements. a, Experimental setups for OER catalysis in pH = 0.25 acid. b, Water electrolysis cell.

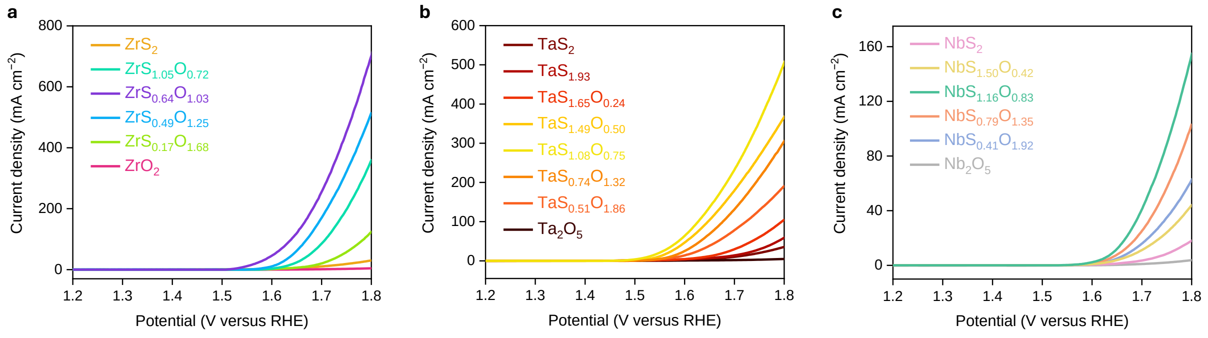

Supplementary Fig. 12 | OER catalysis in pH ≈ 0 acid. a, Polarization curves of ZrS_2, ZrS_xO_y and ZrO_2. b, Polarization curves of TaS_2, TaS_xO_y and Ta_2O_5. c, Polarization curves of NbS_2, NbS_xO_y and Nb_2O_5.

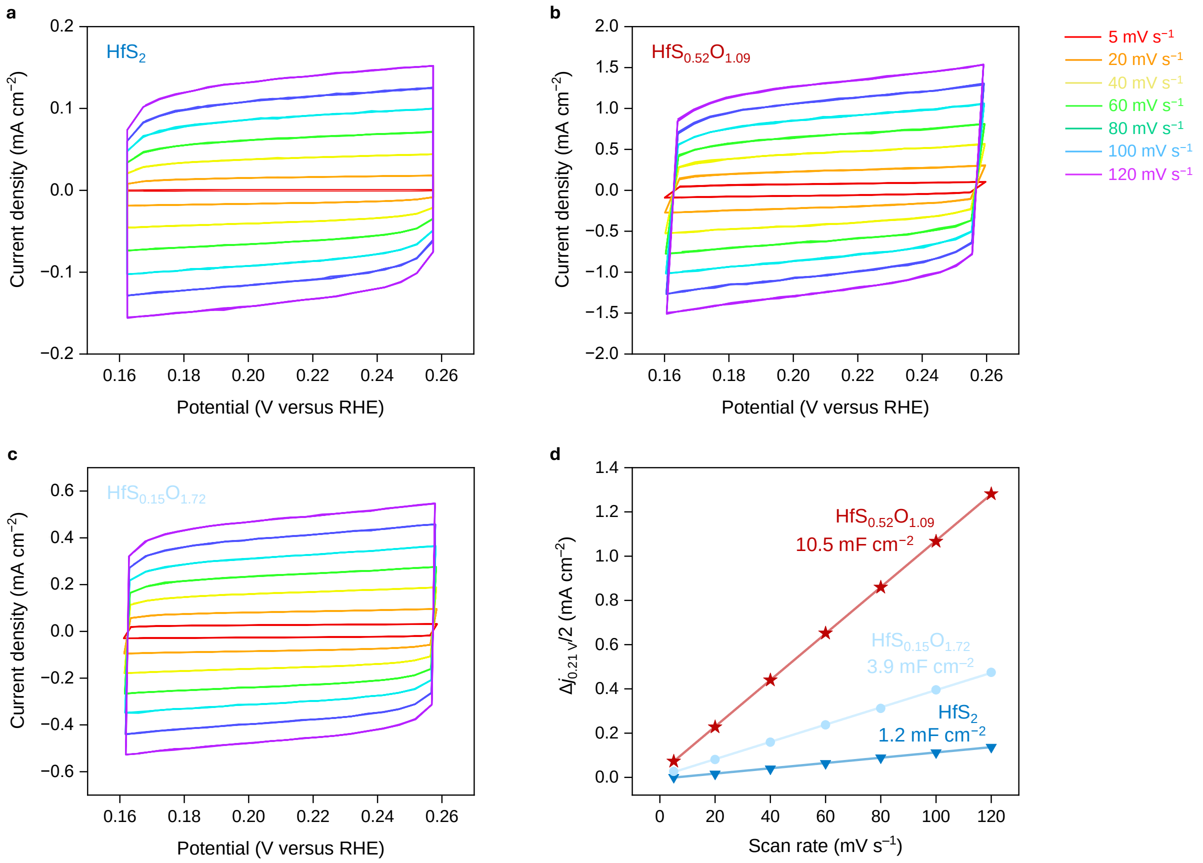

Supplementary Fig. 13 | Investigation of C_{dl}. a–c, CV curves of HfS_2 (a), $HfS_{0.52}O_{1.09}$ (b) and $HfS_{0.31}O_{1.48}$ (c), measured in the potential window of 160–260 mV versus RHE. The scan rates vary from 5 to 120 mV s^{-1}. d, Plots derived from (a–c), showing half of the current differences at +0.21 V versus RHE as a function of scan rate. The linear fits indicate the C_{dl} of the catalysts.

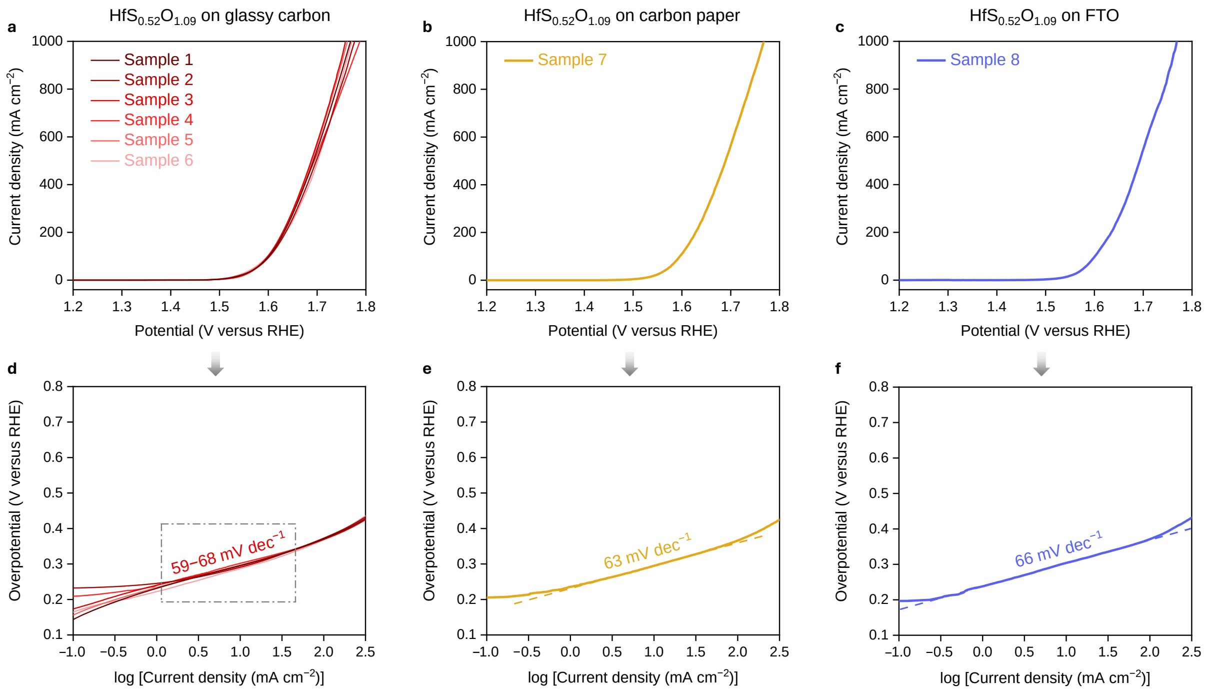

Supplementary Fig. 14 | Reproducibility of OER measurements. a–c, Polarization curves and b–d, the corresponding Tafel slopes of $HfS_{0.52}O_{1.09}$ catalysts that are supported by glassy carbon (a,d), carbon paper (b,e) and FTO (c,f). Multiple samples were tested using glassy carbon as the support (a), which exhibit slight variations in the electrocatalytic performance. The grey box in (d) shows where the Tafel slopes were obtained.

We correlate the OER activities of the Hf-based catalysts with their structures by density functional theory (DFT) calculations. Since the basal planes of HfS_2 without exposed Hf atoms cannot adsorb reactive species, we focus on the edge sites of HfS_2 nanosheets (Supplementary Fig. 15a) and calculate the overpotential for OER at pH = 0 (Supplementary Fig. 15b). The overpotential of pristine HfS_2 [Supplementary Fig. 15a(1)] is 2.69 eV. As indicated by the HAADF-STEM and X-ray spectroscopy results (Fig. 2), S vacancies and O substitutions are considered. We found that the overpotential is reduced to 0.30 eV with an increasing number

of S vacancies and O substitutions [Supplementary Fig. 15a(2−6)], close to the values of 0.295 ± 0.007 eV obtained from OER measurements (Supplementary Fig. 14a−c). However, further increases in S vacancies and O substitutions [Supplementary Fig. 15a(7,8)] lead to higher overpotentials. These verify the enhancement and optimization of catalytic activities through the evolution of structural defects.

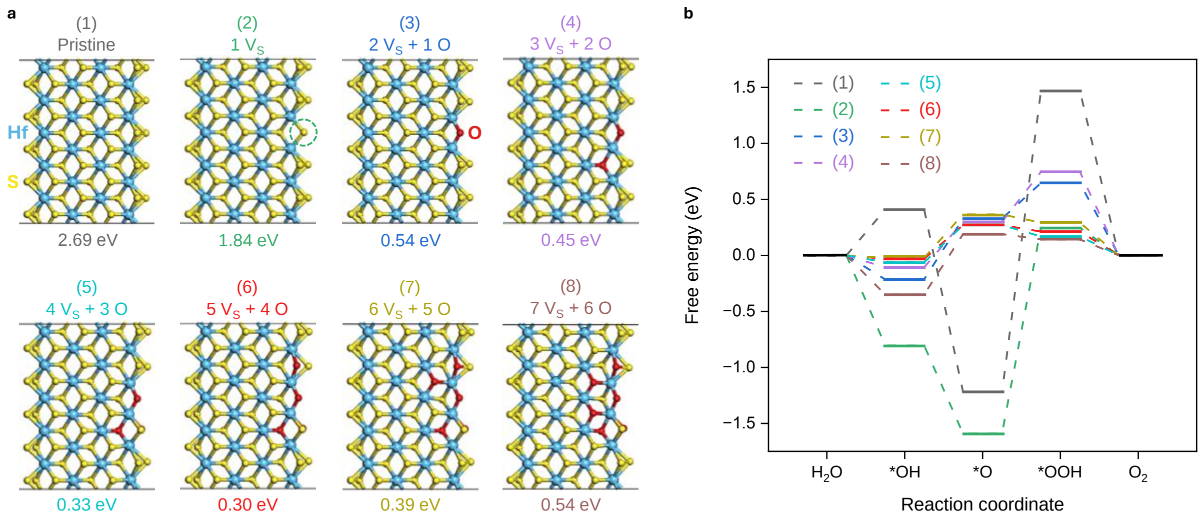

Supplementary Fig. 15 | DFT calculations. a, Atomic models of (1) pristine HfS_2, (2) HfS_2 with one S vacancy (V_S, green circle), and (3−8) HfS_2 with increasing S vacancies and O substitutions. b, Gibbs free energy diagram for the structures illustrated in (a) for OER electrocatalysis at pH = 0.

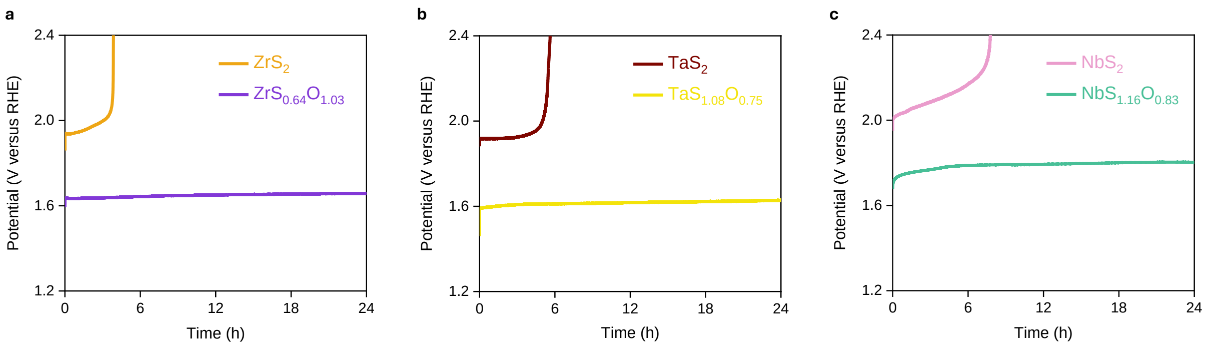

Supplementary Fig. 16 | Stability measurements of TMOCs and TMDs for OER at pH ≈

0. a, Chronopotentiometric tests of ZrS_2 and $ZrS_{0.64}O_{1.03}$. b, Chronopotentiometric tests of TaS_2 and $TaS_{1.08}O_{0.75}$. c, Chronopotentiometric tests of NbS_2 and $NbS_{1.16}O_{0.83}$. The TMOCs are more stable compared with their patent TMDs.

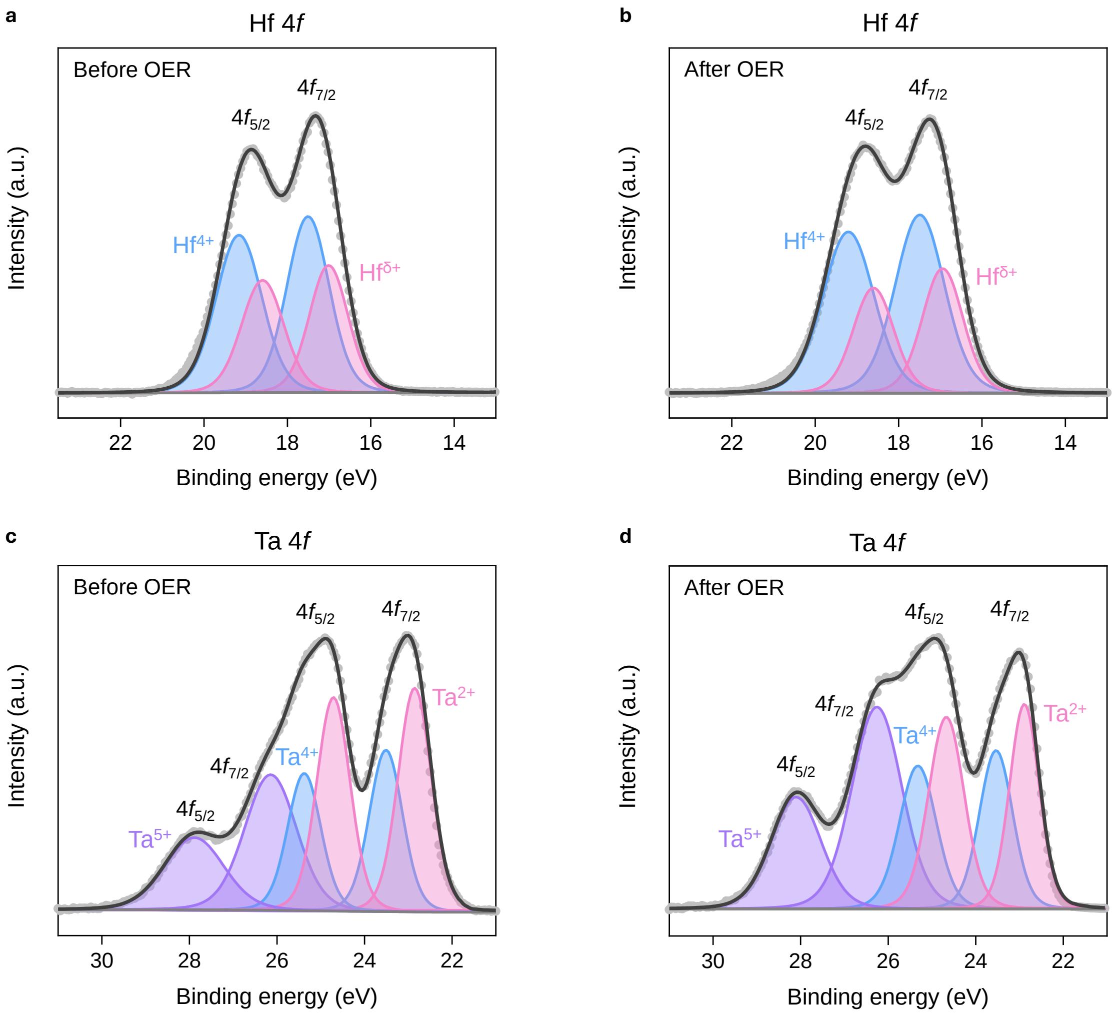

Supplementary Fig. 17 | XPS before and after OER measurements. a,b, Hf 4*f* core-level spectra for $HfS_{0.52}O_{1.09}$ and c,d, Ta 4*f* core-level spectra for $TaS_{1.08}O_{0.75}$ before (a,c) and after (b,d) the stability tests, where the current density was maintained at 20 mA cm^{-2} for 120 hours. The oxygen content of Hf-based catalyst was slightly increased from 67.7% ($HfS_{0.52}O_{1.09}$) into 69.3% ($HfS_{0.50}O_{1.13}$). The oxygen content of Ta-based catalyst was increased from 41.0%

($TaS_{1.08}O_{0.75}$) into 47.8% ($TaS_{0.97}O_{0.89}$).

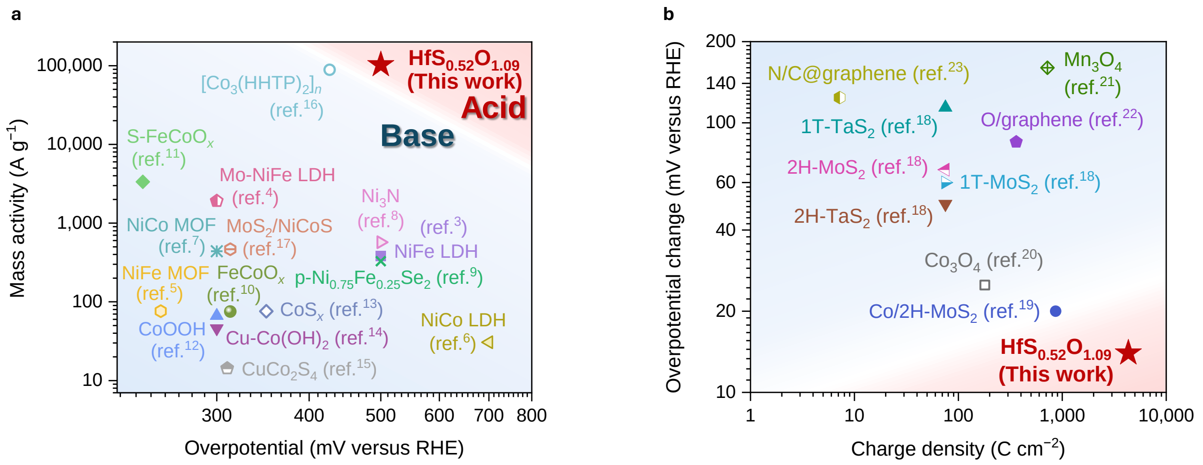

Supplementary Fig. 18 | Comparison of OER performances with literature. a, Comparison of mass activity of $HfS_{0.52}O_{1.09}$ at pH = 0.25 with other rare-metal-free ultrathin OER catalysts at pH = 13 or 14. b, Comparison of stability (overpotential change versus charge density) of nanosheet catalysts at pH ≈ 0.

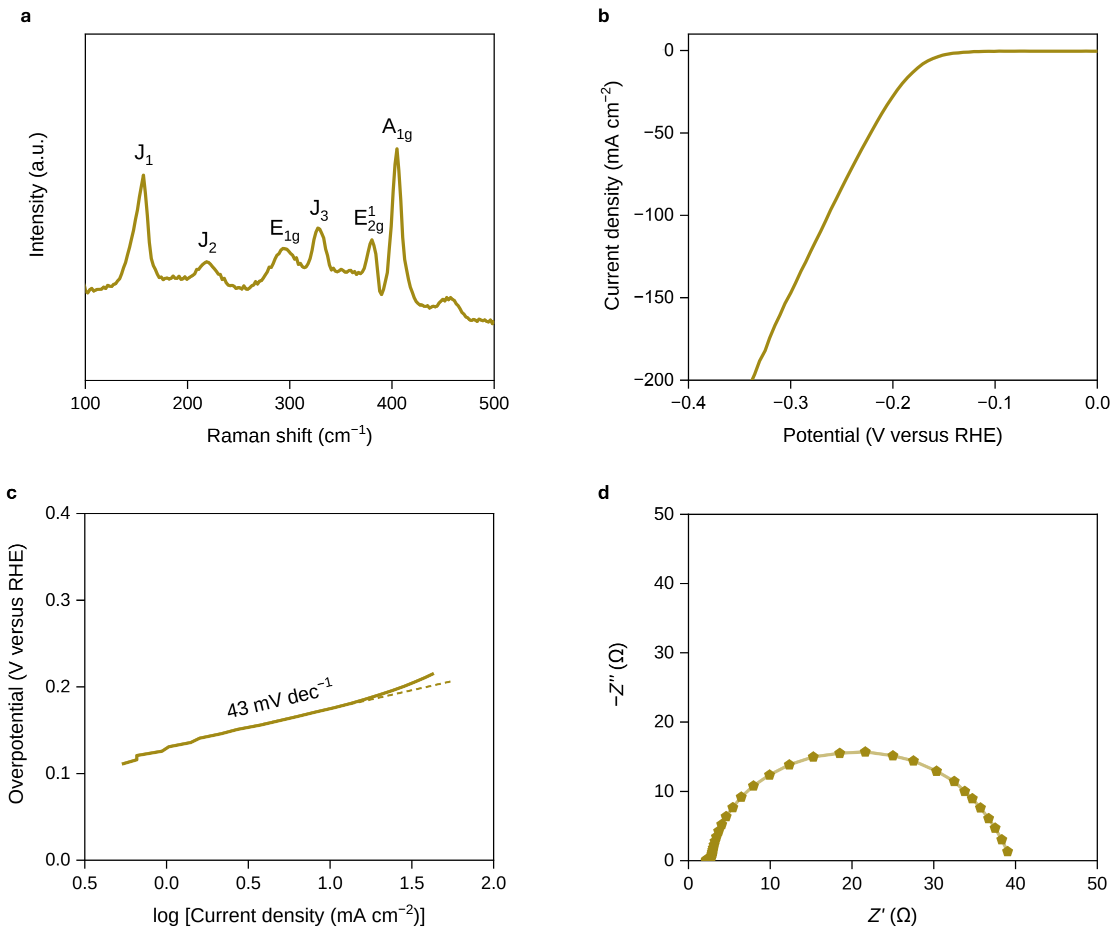

Supplementary Fig. 19 | Characterization and electrochemical measurements of 1T-MoS2.

a, Raman spectrum of 1T-MoS2. b, Polarization curves with *iR* correction for HER at pH = 0.25. c, Tafel slopes acquired from (b). d, Nyquists plots obtained by EIS.

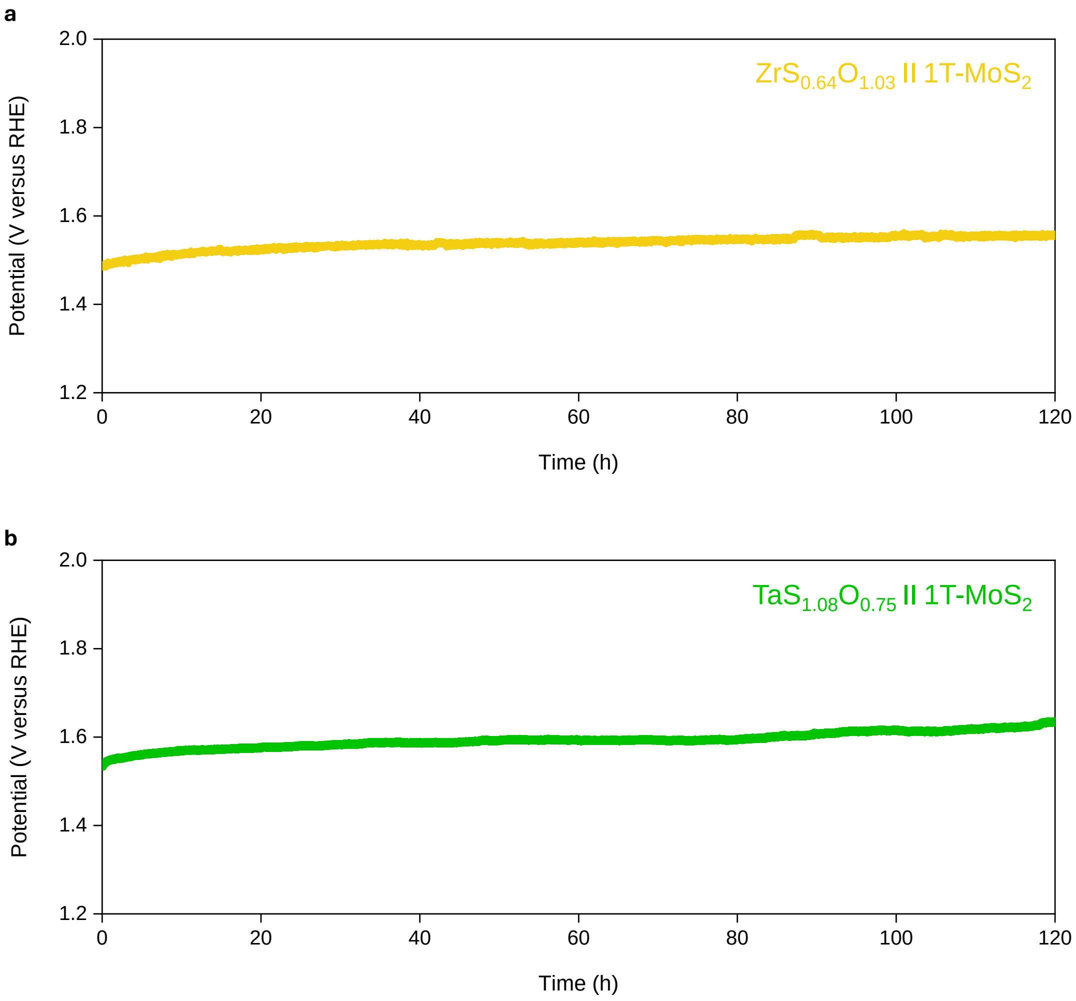

Supplementary Fig. 20 | Stability measurements of PEM water electrolysers. Chronopotentiometric tests of a, $ZrS_{0.64}O_{1.03}$ || 1T-MoS_2 and b, $TaS_{1.08}O_{0.75}$ || 1T-MoS_2 electrolysers at a current density of 20 mA cm^{-2}.

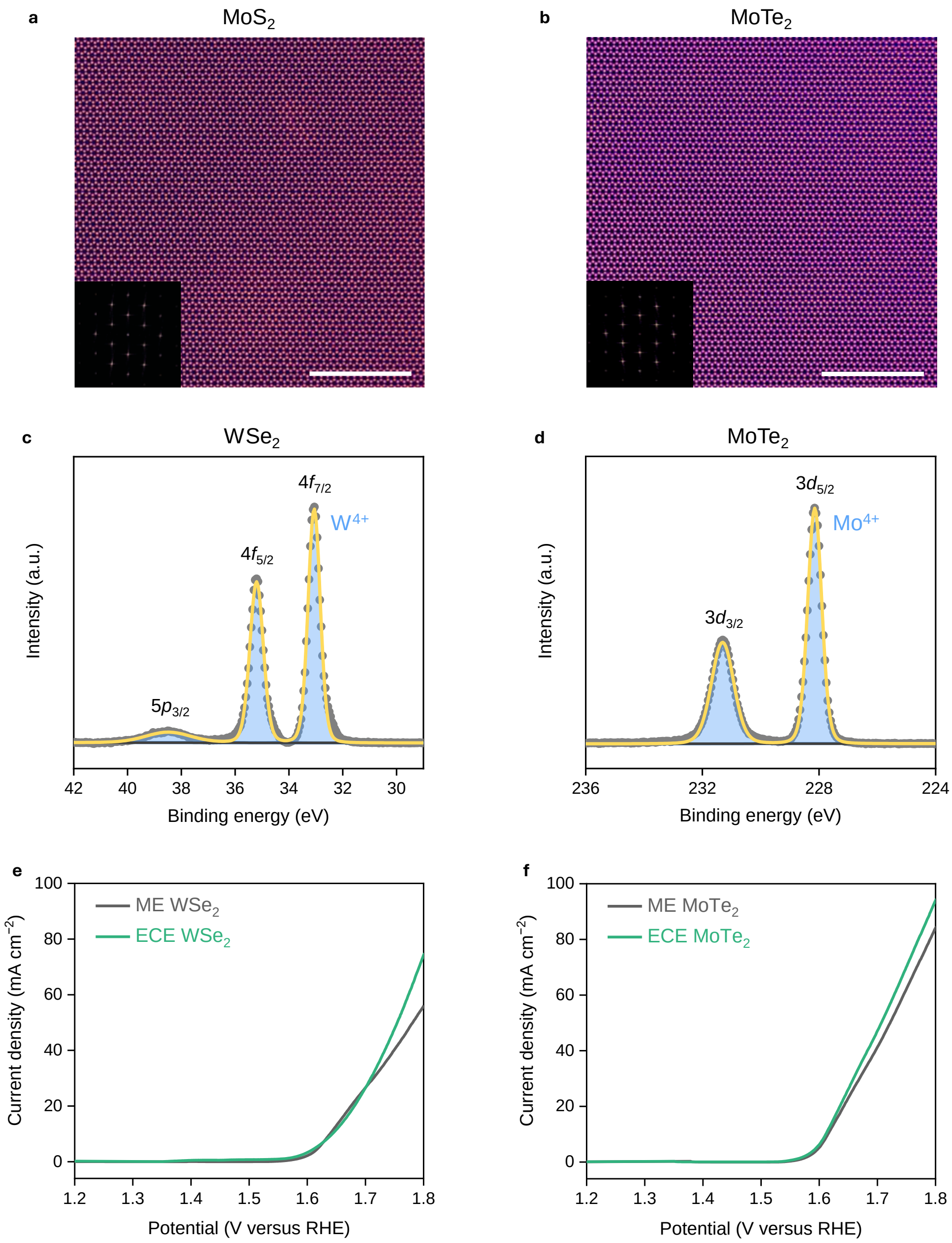

Supplementary Fig. 21 | Exfoliated group 6 TMDs. a,b, HAADF-STEM images of electrochemically exfoliated MoS_2 (a) and $MoTe_2$ (b) nanosheets using TBA^+ intercalation. Scale bars: 5 nm. Insets: Corresponding FFT patterns. It can be seen that MoS_2 and $MoTe_2$ retain high crystallinity with few defects after the electrochemical exfoliation. c, W 4*f* core-level XPS spectrum of electrochemically exfoliated WSe_2 nanosheets. d, Mo 3*d* core-level XPS spectrum of electrochemically exfoliated $MoTe_2$ nanosheets. The W and Mo reside in +4 states,

the same as in their parent disulphides. These results indicate negligible exposure of W/Mo atoms (which would otherwise lead to decreased chemical states) or oxidation of W/Mo atoms (which would otherwise lead to increased chemical states). By comparing with groups 4 and 5 TMDs, we found that the oxidation stemming from electrochemical exfoliation may be dependent on the group number of the transition metals and group 6 TMOCs are unlikely to be obtained using such synthesis method. e,f, Polarization curves of electrochemically exfoliated (ECE) WSe_2 (e) and $MoTe_2$ (f) nanosheets for OER catalysis in pH ≈ 0 acid, with their mechanically exfoliated (ME) counterparts as references. The slight differences in the catalytic activity suggest that the W and Mo are not activated through the TBA^+ intercalation.

Supplementary Table 1 | Fitting parameters for EXAFS of $HfS_{0.73}O_{0.96}$. *N*: Coordination number, *R*: bond length, σ^2: Debye-Waller factor, namely, mean square relative displacement owing to atom vibrations.

Path	*N*	*R* (Å)	σ^2
Hf−S	2.5	2.78	0.004
Hf−O	3.3	2.16	0.002

The amplitude factor (S_0^2) was fixed at 1. The energy correction (ΔE_0) was varied as a global fit parameter, returning a value of (−6 ± 1) eV. The data range was $1.0 \leq R \leq 6.0$ Å, and the misfit (*R*-factor) was 1.9%.

Supplementary Table 2 | Fitting parameters for EXAFS of $HfS_{0.52}O_{1.09}$.

Path	*N*	*R* (Å)	σ^2

Hf−S	1.9	2.72	0.005
Hf−O	4.0	2.12	0.003

The S_0^2 was fixed at 1. The ΔE_0 was varied as a global fit parameter, returning a value of (−7 ± 2) eV. The data range was $1.0 \leq R \leq 6.0$ Å, and the R-factor was 1.5%.

Supplementary Table 3 | Fitting parameters for EXAFS of $HfS_{0.31}O_{1.48}$.

Path	N	R (Å)	σ^2
Hf−S	1.3	2.63	0.004
Hf−O	6.2	2.05	0.001

The S_0^2 was fixed at 1. The ΔE_0 was varied as a global fit parameter, returning a value of (−8 ± 3) eV. The data range was $1.0 \leq R \leq 6.0$ Å, and the R-factor was 2.3%.

Supplementary Table 4 | Determination of the stoichiometric ratio of HfS_xO_y.

Material	XPS	EXAFS
$HfS_{0.73}O_{0.96}$	Hf^{2+}:Hf^{4+} = 1:2.23, Hf:(S + O) = 1:1.69	Hf−S:Hf−O = 1:1.32
$HfS_{0.52}O_{1.09}$	Hf^{2+}:Hf^{4+} = 1:1.56, Hf:(S + O) = 1:1.61	Hf−S:Hf−O = 1:2.10
$HfS_{0.31}O_{1.48}$	Hf^{2+}:Hf^{4+} = 1:3.76, Hf:(S + O) = 1:1.79	Hf−S:Hf−O = 1:4.77

Supplementary Table 5 | Electrochemical exfoliation conditions for synthesizing TMOC catalysts. The parameters shown here resulted in optimized activities for OER at pH ≈ 0.

TMOC	Potential	Temperature	TBA^+ concentration

	(V)	(°C)	(mM)
$HfS_{0.52}O_{1.09}$	−5.0	20	7.5
$ZrS_{0.64}O_{1.03}$	−4.6	20	7.5
$TiS_{0.75}O_{0.91}$	−3.5	50	0.5
$TaS_{1.08}O_{0.75}$	−2.8	100	0.2
$NbS_{1.16}O_{0.83}$	−4.0	80	1
$VSe_{1.04}O_{1.27}$	−4.5	65	10
$HfSe_{0.31}O_{1.40}$	−7.2	20	20
$ZrSe_{0.36}O_{1.28}$	−6.5	20	20
$TaTe_{0.72}O_{1.54}$	−6.0	45	5

Supplementary Table 6 | Literature survey on mass activity of rare-metal-free ultrathin electrocatalysts. For each system, the best result is presented.

Material	Electrolyte (pH)	Overpotential (mV versus RHE)	Mass activity (A g^{-1})	Reference
$HfS_{0.52}O_{1.09}$	0.25	500	102,959 ± 7416	This work
NiFe LDH	14	500	382.8	[3]
Mo-NiFe LDH	14	300	1910	[4]
NiFe MOF	14	252	76.07	[5]
NiCo LDH	13	700	30.6	[6]
NiCo MOF	14	300	440	[7]

Ni_3N	14	500	572	8
p-$Ni_{0.75}Fe_{0.25}Se_2$	14	500	328.19	9
$FeCoO_x$	13	350	54.9	10
S-$FeCoO_x$	14	270	2440	11
CoOOH	14	300	66.6	12
CoS_x	14	350	76	13
Cu-$Co(OH)_2$	14	300	46	14
$CuCo_2S_4$	14	310	14.29	15
$[Co_3(HHTP)_2]_n$	13	470	64,630	16
MoS_2/NiCoS	14	350	340	17

LDHs represent layered double hydroxides. HHTP is 2,3,6,7,10,11-hexahydroxytriphenylene. The prefixes indicate the electronic or elemental doping. The slash means that it is a heterostructure.

Supplementary Table 7 | Literature survey on chronopotentiometric stability of rare-metal-free ultrathin electrocatalysts for OER at pH ≈ 0.

Material	Overpotential change (mV versus RHE)	Current density (mA cm^{-2})	Time (hour)	Reference
$HfS_{0.52}O_{1.09}$	14 ± 3	50	24	This work
2H-MoS_2	67	10	2.1	18
1T-MoS_2	60	10	2.1	18
2H-TaS_2	50	10	2.1	18

1T-TaS_2	114	10	2.1	[18]
Co/2H-MoS_2	20	10	24	[19]
Co_3O_4	25	1	50	[20]
Mn_3O_4	160	10	20	[21]
O/graphene	85	10	10	[22]
N/C@graphene	124	1	2	[23]

The prefixes indicate the phases of the materials. The elements before the slashes show the functionalization or doping agents.

References

1. Makuła, P., Pacia, M. & Macyk, W. How to correctly determine the band gap energy of modified semiconductor photocatalysts based on UV−vis spectra. *J. Phys. Chem. Lett.* **9**, 6814–6817 (2018).
2. Wang, D. *et al.* Selective direct growth of atomic layered HfS_2 on hexagonal boron nitride for high performance photodetectors. *Chem. Mater.* **30**, 3819–3826 (2018).
3. Zhang, K., Wang, W., Kuai, L. & Geng, B. A facile and efficient strategy to gram-scale preparation of composition-controllable Ni-Fe LDHs nanosheets for superior OER catalysis. *Electrochim. Acta* **225**, 303–309 (2017).
4. He, Z. *et al.* Activating lattice oxygen in NiFe-based (oxy)hydroxide for water electrolysis. *Nat. Commun.* **13**, 2191 (2022).
5. Sun, F. *et al.* NiFe-based metal–organic framework nanosheets directly supported on nickel foam acting as robust electrodes for electrochemical oxygen evolution reaction.

Adv. Energy Mater. **8**, 1800584 (2018).

6. Jiang, J., Zhang, A., Li, L. & Ai, L. Nickel–cobalt layered double hydroxide nanosheets as high-performance electrocatalyst for oxygen evolution reaction. *J. Power Sources* **278**, 445–451 (2015).
7. Huang, L. *et al.* Self-dissociation-assembly of ultrathin metal-organic framework nanosheet arrays for efficient oxygen evolution. *Nano Energy* **68**, 104296 (2020).
8. Xu, K. *et al.* Metallic nickel nitride nanosheets realizing enhanced electrochemical water oxidation. *J. Am. Chem. Soc.* **137**, 4119–4125 (2015).
9. Huang, Y., Jiang, L. W., Shi, B. Y., Ryan, K. M. & Wang, J. J. Highly efficient oxygen evolution reaction enabled by phosphorus doping of the Fe electronic structure in iron–nickel selenide nanosheets. *Adv. Sci.* **8**, 2101775 (2021).
10. Zhuang, L. *et al.* Ultrathin iron-cobalt oxide nanosheets with abundant oxygen vacancies for the oxygen evolution reaction. *Adv. Mater.* **29**, 1606793 (2017).
11. Zhuang, L. *et al.* Sulfur-modified oxygen vacancies in iron–cobalt oxide nanosheets: enabling extremely high activity of the oxygen evolution reaction to achieve the industrial water splitting benchmark. *Angew. Chem. Int. Ed.* **59**, 14664–14670 (2020).
12. Huang, J. *et al.* CoOOH nanosheets with high mass activity for water oxidation. *Angew. Chem.* **127**, 8846–8851 (2015).
13. Ju, S. *et al.* In situ surface chemistry engineering of cobalt-sulfide nanosheets for improved oxygen evolution activity. *ACS Appl. Energy Mater.* **2**, 4439–4449 (2019).
14. Chen, L. *et al.* Facile synthesis of Cu doped cobalt hydroxide (Cu–$Co(OH)_2$) nano-sheets for efficient electrocatalytic oxygen evolution. *J. Mater. Chem. A* **5**, 22568–22575 (2017).

15. Chauhan, M., Reddy, K. P., Gopinath, C. S. & Deka, S. Copper cobalt sulfide nanosheets realizing a promising electrocatalytic oxygen evolution reaction. *ACS Catal.* **7**, 5871–5879 (2017).

16. Zhang, M. *et al.* Fewer-layer conductive metal-organic nanosheets enable ultrahigh mass activity for the oxygen evolution reaction. *Chem. Commun.* **54**, 13579–13582 (2018).

17. Qin, C. *et al.* Interface engineering: Few-layer MoS_2 coupled to a NiCo-sulfide nanosheet heterostructure as a bifunctional electrocatalyst for overall water splitting. *J. Mater. Chem. A* **7**, 27594–27602 (2019).

18. Wu, J. *et al.* Exfoliated 2D transition metal disulfides for enhanced electrocatalysis of oxygen evolution reaction in acidic medium. *Adv. Mater. Interfaces* **3**, 1500669 (2016).

19. Wei, Q. *et al.* Fabrication of Co doped MoS_2 nanosheets with enlarged interlayer spacing as efficient and pH-Universal bifunctional electrocatalyst for overall water splitting. *Ceram. Int.* **47**, 24501–24510 (2021).

20. Mondschein, J. S. *et al.* Crystalline cobalt oxide films for sustained electrocatalytic oxygen evolution under strongly acidic conditions. *Chem. Mater.* **29**, 950–957 (2017).

21. Zhao, Z. *et al.* Tailoring manganese oxide nanoplates enhances oxygen evolution catalysis in acid. *J. Catal.* **405**, 265–272 (2022).

22. Lei, C. *et al.* High-performance metal-free nanosheets array electrocatalyst for oxygen evolution reaction in acid. *Adv. Funct. Mater.* **30**, 2003000 (2020).

23. Sun, J. *et al.* Ultrathin nitrogen-doped holey carbon@graphene bifunctional electrocatalyst for oxygen reduction and evolution reactions in alkaline and acidic media. *Angew. Chem. Int. Ed.* **57**, 16511–16515 (2018).
